# A care quality dashboard for general practitioners managing patients with diabetes mellitus type 2: user-centered design and prototype evaluation

**DOI:** 10.1186/s12911-026-03492-3

**Published:** 2026-05-09

**Authors:** Lola Jo Ackermann, Odile-Florence Giger, Marinja Principe, Michael Brändle, Mia Jovanova, Tobias Kowatsch

**Affiliations:** 1 School of Medicine, University of St.Gallen, St.Gallen, Switzerland; 2 Institute of Technology Management, University of St.Gallen, St.Gallen, Switzerland; 3 Institute for Implementation Science in Health Care, University of Zurich, Zürich, Switzerland; 4 HOCH Health Ostschweiz, Cantonal Hospital St.Gallen, St.Gallen, Switzerland; 5 Department of Management, Technology and Economics, ETH Zurich, Zurich, Switzerland

**Keywords:** Diabetes mellitus type 2, User-centered design, Prototype, Dashboard, Qualitative methods, Interviews, Switzerland, SGED score

## Abstract

**Background:**

Diabetes mellitus type 2 (T2D) is a growing burden in Switzerland, where general practitioners (GPs) face increasing workload. To evaluate the quality of T2D care, the Swiss Society of Endocrinology and Diabetology (SGED) developed the SGED score to help GPs overview aggregated patient parameters at the practice level. However, the practical use of the SGED score is hampered by paper-based workflows and fragmented documentation. Currently, no dashboard exists to specifically visualize the SGED score, which overviews aggregated population parameters such as HbA1c or blood pressure. To address this gap, this study examined: (1) what functional requirements healthcare professionals consider essential for such a potential SGED dashboard, and (2) how do healthcare professionals evaluate the usability and clinical relevance of an iteratively developed dashboard prototype.

**Methods:**

We employed an iterative, user-centered three-step approach involving 10 semi-structured interviews with 14 Swiss T2D healthcare professionals. Step 1 involved defining the project scope, identifying predefined functional requirements, and developing an initial SGED score dashboard prototype. Step 2 collected user-generated requirements and prioritized all requirements using the “Must Have”, “Should Have”, “Could Have”, “Won’t Have” (MoSCoW) method. In step 3, the high-fidelity Figma dashboard prototype was iteratively refined based on the requirements and interviewee feedback.

**Results:**

Key functional requirements of the digital SGED score included reminder and alert functions for missing or overdue SGED-relevant assessments, color-coded critical values such as low nephropathy screening rates, demographic overviews, trend analyses of SGED indicators at practice level, benchmarking within practice networks, and exportable reports. Additional needs emerged for patient-level views, integrated checklists, inclusion of comorbidities, and personal or practice-specific goal-setting features. Iterative refinements based on user feedback improved clarity, usability, and visual appeal. Some participants highlighted the dashboard’s intuitive design, clear and diverse visualizations, and benchmarking functionalities, describing it as both engaging and efficient. Others raised concerns about limited suitability for daily clinical workflows, potential integration challenges with existing systems, and the need for interactive, patient-centered features to support routine care.

**Conclusion:**

The proposed SGED score dashboard could enhance T2D care through features like population overviews, long-term visualizations, and anonymized benchmarking, meaning the ability to compare a practice’s SGED performance with those of other practices. Successful clinical adoption will heavily depend on interoperability and seamless integration into existing workflows. The identified requirements provide a foundation for future digital T2D management systems.

**Supplementary Information:**

The online version contains supplementary material available at 10.1186/s12911-026-03492-3.

## Background

Diabetes mellitus type 2 (T2D) presents an escalating public health challenge, placing a growing burden on healthcare systems worldwide. Projections suggest that by 2050, the number of individuals living with T2D will reach over 640 million, with more than one in three adults aged 20–79 in Western countries affected [1]. Switzerland reflects these global trends, making it crucial to address associated health and economic T2D challenges. In Switzerland, general practitioners (GPs) play a key role in T2D management by serving as the first line of defense in monitoring patients with T2D [2]. However, GPs face increasing workload pressures, while the number of practicing GPs is declining [3, 4]. These conditions have been linked to difficulties in consistently providing high-quality care for T2D patients, undermining care quality [5].

To this end, the Swiss Society of Endocrinology and Diabetology (SGED) developed a descriptive primary care quality monitoring overview, the SGED score, to evaluate how effectively GP practices managed their respective T2D populations. The score is based on a set of key clinical indicators that GPs report during routine T2D patient visits. Specifically, GPs are asked to track eight individual-level indicators: general diabetes control (regular visits), body mass index, nicotine use, HbA1c, blood pressure, LDL cholesterol, nephropathy screening, retinopathy screening, and foot examination [6]. These eight indicators were selected based on national and international diabetes management guidelines, adapted to the Swiss healthcare context [7]. The selection focused on parameters that are clinically relevant and routinely measurable in primary care, as well as screening for common diabetes-associated complications.

The SGED score is calculated at the overall practice level by aggregating individual patient-level parameters. A point-based system is used in which practices receive points when predefined population-level targets for each indicator are achieved. The maximum possible score is 100 points. The recommended aim is to achieve ≥ 70/100 points per practice, which was defined by the SGED working group as a realistic and adequate benchmark for appropriate T2D care in Swiss primary care settings [7]. This threshold reflects the expectation that the at least 70% of T2D patients within a practice meet guideline-based standards of care.

Despite its clinical relevance, the score exists either only in paper-based form or requires additional manual documentation in secondary tools [8], such as BlueCCM provided by BlueCare, a chronic care indicator tool for managing T2D patients [9]. Given that data required for calculating the SGED score are obtained from routinely documented clinical parameters in individual patient records, the SGED score calculation typically requires manual extraction and documentation, with no automated data integration is currently available [8]. As such, current practices to document the SGED score are likely to increase administrative burden and introduce risks of manual error. Further, the paper-based form prevents continuous tracking of patient progress or evaluation of care quality or treatment effectiveness over time [10, 11]. At present, no digital dashboard exists to enable a comprehensive visualization of the SGED score [8].

To address this gap, we systematically derived functional requirements for a potential digital SGED score dashboard in collaboration with healthcare professionals, building on previous stakeholder analysis in T2D care in Switzerland [8]. Such requirement engineering represents an early stage of the software development cycle [12], ensuring that the resulting system aligns with user needs and clinical workflows, in this case, primarily those of healthcare professionals such as GPs. Based on these requirements, an iterative design process was conducted to develop and evaluate a high-fidelity dashboard prototype to visualize the SGED score. Prototyping represents a crucial phase in product development, facilitating early validation of design concepts [13] and supporting subsequent adoption and integration into routine clinical practice. Guided by principles of user-centered design [14, 15], this study examined the following research questions (RQs):

RQ1: What functional requirements do healthcare professionals consider essential for a digital SGED score dashboard in T2D primary care?

RQ2: How do healthcare professionals evaluate the usability and clinical relevance of a digital SGED score dashboard prototype?

## Methods

**Fig. 1:**
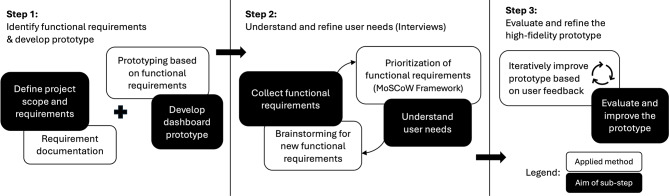
User-centered, iterative three-step approach for prototyping the SGED score dashboard

To address both RQs, we conducted a total of 10 semi-structured interviews with 14 participants (six individual interviews and four group interviews with two participants each), including GPs, Medical Practice Assistants (MPAs), Medical Practice Coordinators (MPCs), and other specialists. We specifically focused on GPs and additional healthcare provides because they play a vital role in T2D management and serve as the primary caregivers for patients, providing essential insights and feedback for the development of a potential SGED score visualization dashboard. All findings are based exclusively on these interviews; no additional interviews were carried out. Each session addressed both RQs sequentially: participants first discussed functional requirements (RQ1) through brainstorming, feedback on predefined requirements, and prioritization, and then evaluated the current version of the dashboard prototype (RQ2). Between sessions, the researcher refined both the consolidated requirements and the prototype based on preceding insights, so that subsequent participants engaged with a progressively updated version of the dashboard (see Fig. 1).

Following established best practices for user-centered design, which emphasize ongoing user involvement to improve usability and fit in real-world settings [15, 16], we adopted an iterative, user-centered three-step approach (see Fig. 1). Step 1 focused on defining the project scope, deriving the preliminary functional requirements from the literature, and developing an initial high-fidelity prototype prior to the interviews. Step 2 was aimed at understanding and refining the user needs in relation to the functional requirements (RQ1), and Step 3 was about evaluating and iteratively refining the prototype based on user feedback (RQ2). Within each interview session, Steps 2 and 3 were addressed sequentially. Between sessions, the iterative refinement of both requirements and the prototype occurred, creating the development cycle depicted in Fig. 1.

### Step 1: Identification of predefined requirements and initial prototype development

In the first step, we defined functional requirements to provide a foundation for the semi-structured interviews, informed by a recent study addressing challenges associated with the SGED score in the Swiss context [8], as well as studies for dashboard requirements for hospitals [17], primary care dashboards [18], and diabetes care [19]. Motivated by literature, these predefined requirements (see Table 1, Additional File 8) were used as a starting point for Step 2 (“Understand and Refine User Needs”) and for the discussion during the interviews. Next, the predefined functional requirements were translated once into a persona, followed by the development of ten user stories and one scenario (see Table 1, Additional File 6). These narrative elements ensured that the design process remained user-centered while also providing a structured basis for prototype development [20]. The requirements were then transformed into an initial high-fidelity prototype of the dashboard using Figma [21], a collaborative web-based design tool used to visualize and prototype user interfaces. This initial prototype was then evaluated and updated across interviews.

**Table 1:** Overview over additional files and their content

File name	File format	Title of data	Description of data
Additional File 1	.docx	Consolidated criteria for reporting qualitative studies using the COREQ (Consolidated Criteria for Reporting Qualitative Research) 32-item checklist	Completed 32-item COREQ checklist used to ensure comprehensive and transparent reporting of qualitative research methods and findings.
Additional File 2	.docx	Study information and consent form	Information sheet and consent form provided to all study participants, outlining the study purpose, procedures, data handling, and participant rights.
Additional File 3	.docx	Semi-structured interview guide	List of guiding questions and topics used during interviews with stakeholders, including general practitioners and other healthcare professionals.
Additional File 4	.docx	Interviewee’s backgrounds	Summary table detailing the professional background, role, and relevant experience of each interviewee (anonymized).
Additional File 5	.docx	Coding Framework	Structured framework describing the themes and thematic codes applied during qualitative data analysis.
Additional File 6	.docx	Narrative elements for the prototype	Collection of narrative components informing the design of the digital diabetes management dashboard prototype.
Additional File 7	.docx	SGED Score Dashboard Prototype in German	Mockups of the digital SGED score dashboard prototype, presented in German.
Additional File 8	.docx	Predefined functional requirements for the dashboard	List of the system’s functional requirements defined prior to the design and implementation phases.
Additional File 9	.docx	Example for the “Must Have”, “Should Have”, “Could Have”, “Won’t Have” (MoSCoW) method prioritization framework	Example illustrating how dashboard features were prioritized according to the MoSCoW framework.

### Step 2: Understand and refine user needs

The second step centered on requirement engineering for a dashboard to visualize the SGED score. A semi-structured interview guide was developed for this purpose (see Table 1, Additional File 3), allowing interviewers to ask unplanned, spontaneous questions when appropriate. The 10 semi-structured interviews were carried out to explore user needs and expectations regarding a potential digital SGED score. Participants were explicitly encouraged to critically reflect on the predefined functional requirements. Furthermore, brainstorming sessions helped participants propose additional functional requirements, later referred to as user-generated requirements. These were captured as individual suggestions and were not disclosed to subsequent interviews. Accordingly, no consensus across interviews was required for documenting a user-generated requirement. All predefined and user-generated requirements were then prioritized using the MoSCoW method, which categorizes them into “Must Have”, “Should Have”, “Could Have”, and “Won’t Have” elements [22]. This task was performed collaboratively during the interviews, allowing participants to actively assign priorities to the requirements based on their relative importance for implementation. Since the MoSCoW method (see Table 1, Additional File 9) produces a nominal scale, all requirements within the same category are considered to have equal priority, without ranking one higher or lower than another within the group [22]. This equal priority approach makes it an easily implementable and favorable prioritization technique [23, 24]. This structured prioritization enabled us to focus on the most essential functionalities while still capturing secondary needs for potential future development.

### Step 3: Evaluate and Refine the High-Fidelity Prototype.

In the final step, the prototype was then evaluated iteratively with interviewees during the interview sessions to gather feedback on usability and effectiveness. Insights from these evaluations informed successive refinements of the prototype, ensuring that the dashboard evolved in alignment with user expectations and practical needs. Hence, when a participant suggested a change, the researcher discussed the suggestion in depth with the participant and included it in the next iteration if it aligned with the dashboard’s intended purpose. No formal consensus threshold was applied, as the aim was rapid user-centered iteration. Through this structured cycle of requirement elicitation, evaluation, and refinements, the dashboard was progressively improved to enhance both usability and contextual relevance.

### Selection and recruitment of interviewees

This study received approval from the institution’s ethics committee and was deemed exempt from formal review (date: October 27, 2024). Interviews were carried out between June and August 2025. Written informed consent was obtained from all participants prior to their involvement in the study (see Table 1, Additional File 2). Participants were initially selected through purposive sampling to include individuals with extensive expertise in the field of T2D management. After a positive response, we asked the participants whether they wished to invite an additional colleague who was also involved in the management of patients with T2D. This approach was chosen to capture complementary professional perspectives within the same practice setting and to facilitate interactive discussion between closely collaborating healthcare professionals. In addition, we applied snowball sampling by asking the initial interviewees at the end of the interview to suggest further participants who met the selection criteria, creating a referral chain [25]. Recruitment concluded once theoretical saturation was reached, defined as the point at which three consecutive interviews failed to produce new insights [26, 27]. Overall, all interviews were conducted online in German, lasted between 29 and 55 min, and were conducted by one researcher, who had no prior relationship with the participants.

### Data collection and data analysis

All interviews were recorded via Microsoft Teams. Initial transcripts were automatically generated by Microsoft Teams and subsequently reviewed, with any missing segments manually completed by revisiting the recordings. Afterward, transcripts were anonymized by assigning a unique identifier to each participant. Data analysis was performed concurrently using ATLAS.ti (Version 25.0.1) [28]. To maintain methodological rigor and ensure transparency, the study adhered to the COREQ (Consolidated Criteria for Reporting Qualitative Research) guidelines (see Table 1, Additional File 1) [29]. The transcripts were analyzed inductively using a thematic analysis [30], allowing themes to emerge directly from participants’ experiences and feedback regarding the SGED score dashboard. The thematic analysis was conducted in German by one researcher.

The unit of analysis varied depending on the analytical objective. For the evaluation of predefined functional requirements (Sect.  3.1.1, RQ1), responses were aggregated at the interview level (*n* = 10) to account for shared discussions and jointly expressed perspectives within group interviews. In contrast, the prioritization of user-generated functional requirements (Sect.  3.1.2, RQ1) and the analysis of prototype feedback (Sect.  3.2, RQ2) were conducted at the individual level (*n* = 14) to capture personal perceptions and experiences. This distinction was made to reflect the collaborative nature of requirement discussions in group settings while preserving individual viewpoints for prioritization and usability feedback.

## Results

A total of 14 individuals participated in 10 interviews, comprising four group interviews with two participants and six individual interviews. The sample included six GPs, two MPAs, three MPCs, and three other physicians, all based in Switzerland. Background information on the interviewees is provided in Table 1, Additional File 4. The resulting coding framework is shown in Table 1, Additional File 5.

### Functional requirements

**Fig. 2:**
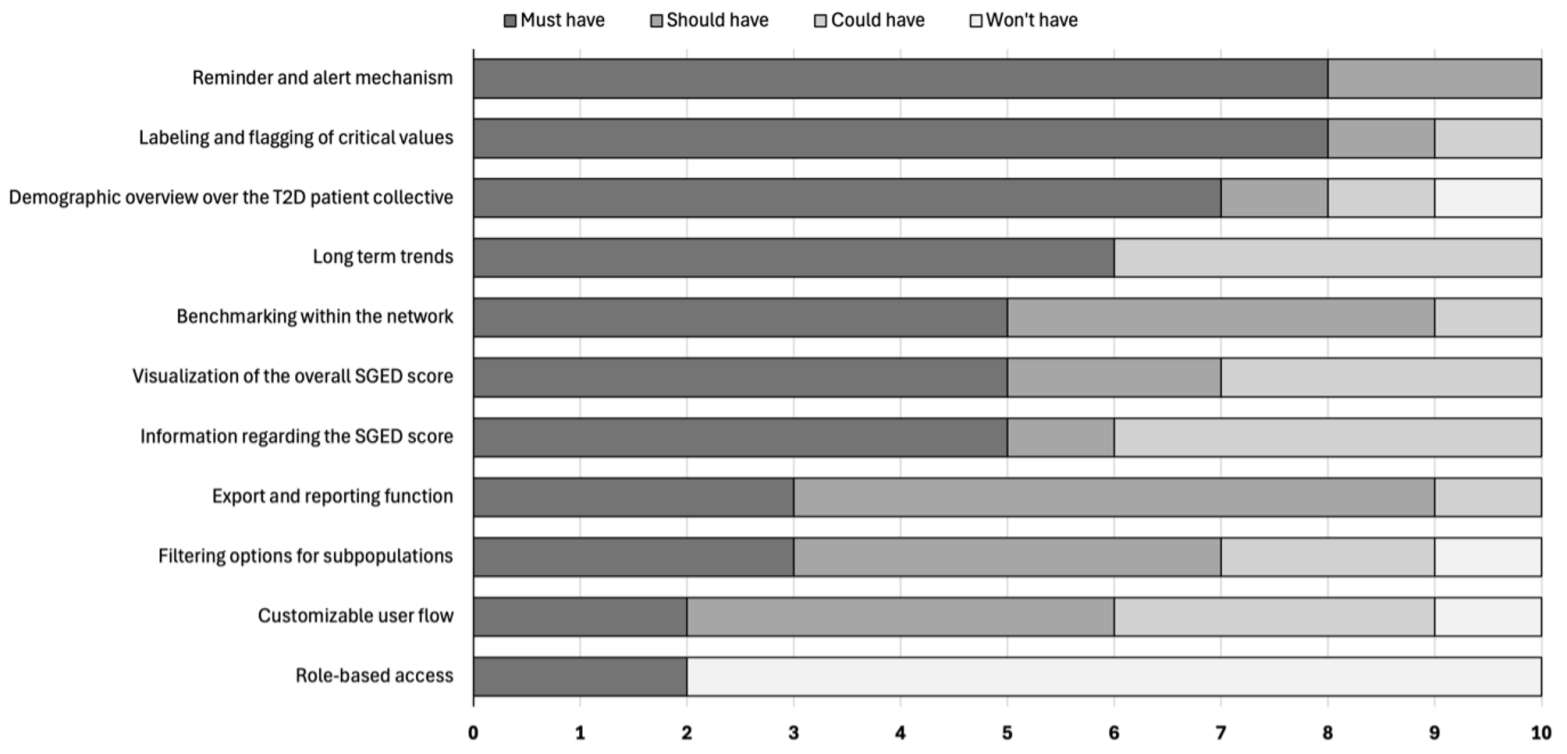
Prioritization of predefined functional requirements by participants using the MoSCoW framework

In this section, we present the functional requirements identified for the visualization dashboard of the SGED score (RQ1), using the MoSCoW framework. These requirements represent the outcome of steps 1 and 2 of the method. They serve to highlight potential elements for the prototype and are derived from the challenges and needs of healthcare professionals involved in the management of T2D. This section is divided into two subparts. Part one focuses on the prioritization of the predefined functional requirements (see Fig. 2), while part two highlights the prioritization of user-generated requirements that emerged from the brainstorming sessions with participants.

#### Predefined functional requirements (see Fig. 2)

Eight out of 10 interviews (8/10) considered a **reminder or alert mechanism** to be essential in a SGED score dashboard. This refers to system-generated notifications indicating missing SGED-relevant data or upcoming assessments, for example when more than 30% of patient records are missing required data. Similarly, eight interviews (8/10) emphasized the need for **labeling and flagging critical values**, such as low nephropathy screening rates, at the aggregate level. This was understood as the visual highlighting of indicators that fall below predefined thresholds, for example through color coding: *“If something is missing in the collective*,* it must be clearly identifiable.”* (P7). While seven (7/10) regarded a **demographic overview of the T2D patient collective**, such as age or gender distribution, as an important element, one (1/10) disagreed with this view. Six (6/10) described **long-term trend analyses** as a “Must Have” feature for evaluating care over time. Here, long-term trend analyses refer to graphical representations of SGED score indicators at the practice level across defined time intervals. The remaining four (4/10) considered such analyses desirable but most definitely not necessary. While half of the interviews (5/10) viewed **benchmarking within the managed care network** as a valuable feature, meaning the ability to compare a practice’s SGED performance with those of other practices: “*It would be exciting if I could compare myself with other family doctors. To see where I stand. That way*,* I could improve.”* (P7), others voiced concerns: “*I don’t actually think it’s a bad thing because it creates transparency*,* but on the other hand*,* I know that it might be a feature that generates the most resistance.”* (P4). Half (5/10) emphasized the importance of **visualizations of the overall SGED score** directly within the dashboard. An **export and reporting function** was perceived by six (6/10) as a “Should Have” feature. This function allows the generation of downloadable reports containing selected aggregated SGED data and visualizations. **Filtering options for subpopulations**, such as specific age groups or gender, were mentioned by three (3/10) as necessary: “*You need to be able to filter and drill down into details if something interests you.”* (P10), by four (4/10) mentioned as important but not essential, whilst one (1/10) criticized that this feature is in no way desirable. Also, one interview (1/10) was completely against the **customizable user flow** of the dashboard. Here, customizable user flow refers to the possibility for general practitioners using the dashboard to individually adapt the structure or navigation sequence of dashboard components, for example adjusting which panels are displayed first. Only two (2/10) categorized it as a “Must Have” feature, making it not considered as critical: *“If it’s piloted well*,* I don’t think it needs to be adjusted individually. Then it should be sufficient as it is.”* (P11). Lastly, opinions differed more strongly regarding **role-based access**, meaning permission-based visibility of dashboard components depending on professional role. While two interviews (2/10) argued in favor of such a feature, the majority (8/10) believed it would be counterproductive: “*For me*,* this goes completely against the principle of care within a multi-professional team.“* (P4).

#### User-generated functional requirements

Four out of fourteen participants (4/14) expressed a desire to **view individual patients** within the visualization dashboard, which currently focuses only on displaying the overall SGED score at the practice level. These four emphasized the importance of accessing patient-specific metrics, typically stored in individual patient records, to better understand and improve population-level outcomes: *“Having an overview of the entire population is good*,* but it’s also important that I can break it down to the individual patient. Because I can only improve the population if I improve the individual.”* (P7). Four interviewees (4/14) highlighted the need for implemented **checklists** to support the systematic documentation of SGED criteria during routine consultations. These checklists should cover all relevant SGED criteria and serve as a practical tool to verify that no essential data or quality indicators are overlooked. They should be accessible directly within the dashboard or downloadable as templates if needed. Several participants emphasized the importance of going beyond the SGED score. For instance, four (4/14) mentioned the need to display **patient comorbidities**, since many patients have additional diseases: *“Things get interesting when different diseases occur together*,* for example*,* hypertension in the context of diabetes or cardiac problems.”* (P7). Three participants (3/14) expressed interest in incorporating data and related to **lifestyle counselling and daily patient-self management**: *“Much of it lies in patient self-management. There are no tools that have action plans that can be given to patients or that have movement logs. There are many protocols out there*,* but ideally they would be unified.”* (P14). Two interviewees (2/14) wished for the inclusion of additional **laboratory data** beyond the SGED score to provide a more detailed clinical overview. Lastly, two (2/14) argued that the recording of **individual goals** per healthcare professional should be possible: *“What might also be exciting is being able to set your own goals. For example: “I want to improve by 50% on this item.” Then you can set it yourself and see: that’s what I set out to do back then. That could serve as a self-motivation.”* (P10). Overall, most of the user-generated functional requirements were identified by GPs rather than by MPAs or MPCs.

### Prototype

This section summarizes the key findings from the user feedback on the prototype dashboard for visualizing the digital SGED score (RQ2). The feedback was collected in open-ended interviews, where participants were not restricted by predefined questions but instead had the freedom to share comments, suggestions, or criticisms based on their own perspectives.

The feedback was integrated into the design through an iterative process. After each interview, the prototype was refined to better align with the expressed preferences and needs of the users. As a result, participants in later interviews were already engaging with a more advanced and tailored version of the dashboard, shaped by the input of earlier users. It should be noted, however, that the current prototype functions purely as a design prototype rather than a fully implemented system. To illustrate the identified functional requirements, mockup data was generated and embedded into the prototype for demonstration purposes. All visualizations have been translated to English; during the interviews, the German version was used (see Table 1, Additional File 7). The visualizations below showcase the most important views of the prototype, implemented based on the defined functional requirements. The cutoff categories for the color-coded legend of the SGED score, displayed across visualizations, were iteratively defined based on user feedback and design considerations in our prototyping process. They do not represent official SGED thresholds but were introduced to structure the visualization into four clearly distinguishable performance levels. Additionally, several views offer alternative visualization formats of the same underlying data (e.g., heatmap, boxplot). To avoid redundancy, only representative examples are shown.

**Fig. 3:**
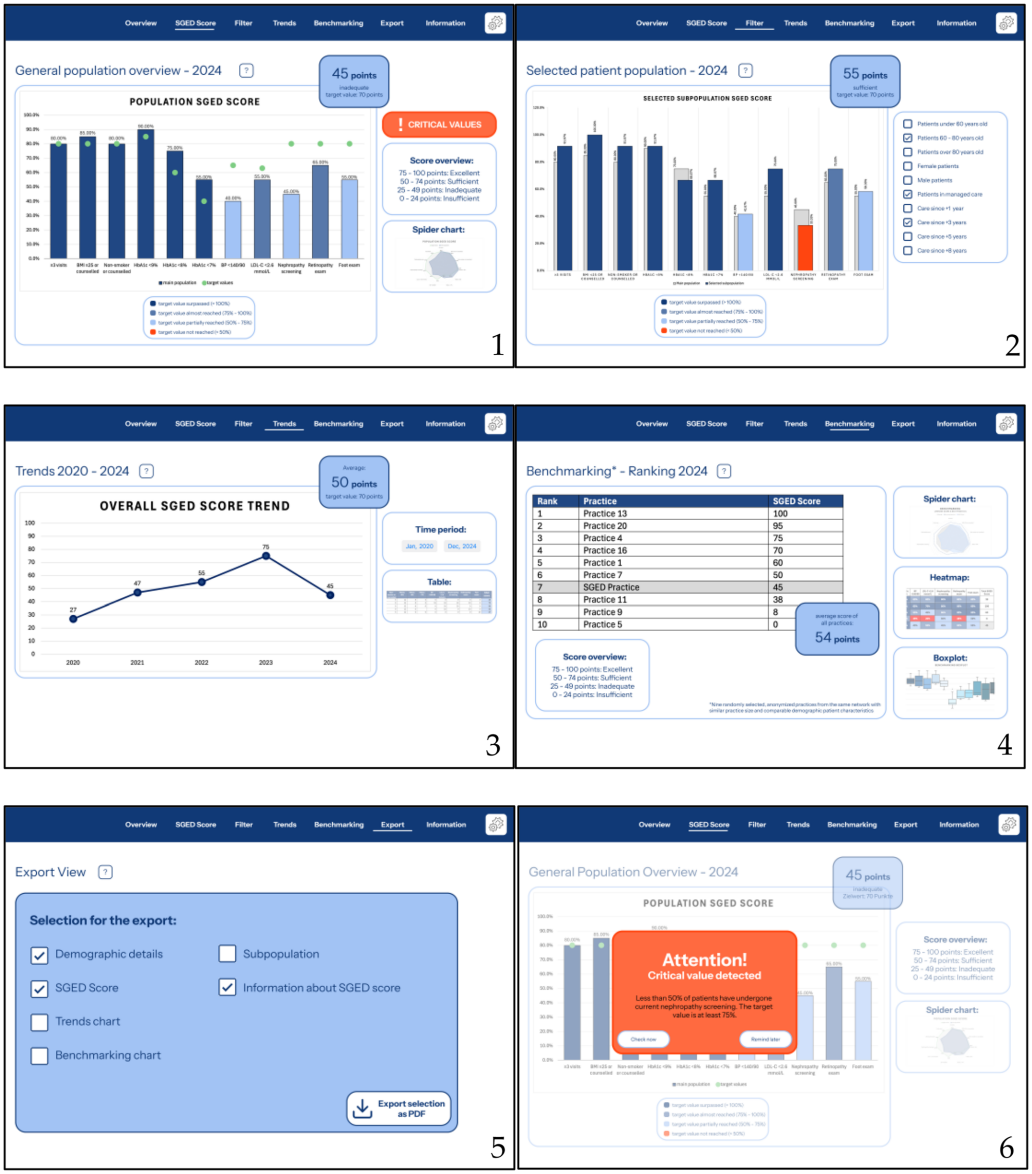
Visualizations of the SGED score dashboard prototype; Panel (1) visualization of the overall SGED score, Panel (2) filtering options for subpopulations, Panel (3) long term trends, Panel (4) anonymized benchmarking within the network, Panel (5) export and reporting function, Panel (6) Reminder and alert mechanism

#### Ease of use and design clarity

Four out of fourteen interviewees (4/14) praised the **clear and straightforward user interface**: *“The design is very appealing*,* clear*,* and simple with just a few clicks. Very effective.”* (P13). This makes the prototype well suited for T2D healthcare professionals, who do not have much time to navigate applications in a clinical setting since they are already under lots of time pressure. Furthermore, four (4/14) expressed their gratitude for the presence and implementation of the **visualizations** regarding the SGED score, since they are informative yet easily understandable. Two (2/14) specifically mentioned that they appreciate the **variety of visualizations** for similar scenarios provided, making it customizable to preferences and exciting to engage: *“I like to play around a bit with different graphics. I find that exciting and helpful.”* (P1). However, **difficulties with interpretation of data** were also common in earlier interviews. Six (6/14) misinterpreted various visualizations due to the absence of appropriate and declarative legends or information pop-ups. Also, the choice of colors in the visualizations occasionally caused confusion, suggesting that a more consistent color scheme would improve clarity.

#### Functional features

Five (5/14) perceived the **benchmarking** within the managed care network (see Fig. 3, Panel 4) as helpful. They appreciated the ability to compare results within the network: “*Even if you don’t do well yourself*,* seeing that everyone else isn’t doing well either can still be a valuable assessment.”* (P3). Participants explained that **benchmarking promotes transparency** and helps identify areas of improvement or recognize when structural factors are hindering effective T2D management. One participant added that not only individual professionals will profit from this, but also entire practices or networks when they analyze and compare the effects of managed care. At the same time, four (4/14) stressed **anonymized benchmarking**: *“Otherwise*,* a bias will arise: only those who are already doing well will participate*,* and a barrier will arise for those who are perhaps not so well-prepared at the moment.”* (P13). Three (3/14) appreciated the **filtering options** (see Fig. 3, Panel 2), as they enable more precise and context-aware evaluations: *“That makes perfect sense. If you want to look at the degree of target achievement for HbA1c*,* for example*,* it varies greatly*,* also depending on age. A patient over 90 is satisfactorily treated with an HbA1c of 8. On the other hand*,* for a 50-year-old type 2 diabetic*,* 7.5 is not yet satisfactory; it is only satisfactory at 6.5 or below.”* (P5). However, two (2/14) wished to have filtering options directly integrated into other views. The specific filter categories were developed based on participant input during the interviews. One (1/14) suggested adding filters to the trends view to directly pinpoint which subpopulation was affected. Another (1/14) proposed including filters in the export function: *“Perhaps it would be useful if you could check boxes for subpopulations again when exporting a file*,* to see directly what belongs to them in a more detailed manner.”* (P10). Four participants (4/14) voiced their interest in viewing **long-term trends** (see Fig. 3, Panel 3), noting that visualizing changes over time helps them track improvements or declines in care quality and detect systemic issues. One (1/14) emphasized that it is important to have adjustable time intervals, allowing users to monitor progress within specific periods. Two (2/14) criticized the **reminder mechanism** (see Fig. 3, Panel 6), describing it as pressuring and unhelpful, since such reminders are rather appropriate in an individual patient context. One (1/14) prefers to identify critical values themselves and therefore does not want to receive constant notifications. Lastly, one (1/14) particularly valued the **export and reporting function** (see Fig. 3, Panel 5), viewing it as a useful tool for sharing visualizations and data with health insurers, showcasing the effectiveness of chronic care within managed care contracts.

#### Clinical relevance and integration

Opinions diverged regarding the prototype’s relevance in everyday clinical practice. Four participants (4/14) voiced overall **positive feedback** and would be interested in using the dashboard in a clinical setting due to potential positive effects on T2D management: *“You can check in from time to time: How are things looking? How do we see ourselves? That’s certainly motivating*,* I think. You see*,* Ah*,* I’ve gotten better here*,* not there*,* we still need to work a bit harder there.”* (P12). Seven (7/14) saw a **limited perceived benefit** and adoption potential in everyday clinical work. They argued that such a visualization dashboard is of limited relevance for routine care, as analyses of the T2D population at the practice-level are only occasionally needed. Six of these (6/14) emphasized the absence of **individual patient overviews**, noting that daily practice focuses primarily on patient-specific treatment decisions: *“When it comes to the population perspective*,* you might look at the overall data once a year. But in a regular doctor’s office*,* I don’t just go by the score. I need a patient cockpit for that.”* (P8). These six (6/14) suggested that, given the limited time available, the healthcare professionals should rather dedicate their time to direct patient interactions. Similarly, three (3/14) were concerned that the dashboard could increase **administrative workload**, which could hinder its adoption. In addition, five (5/14) criticized the current prototype for its **limited flexibility and lack of interactivity**, describing it as too rigid for practical use. They highlighted that it only displays existing data without the possibility to record or adjust data directly within the system. Four (4/14) are also conflicted about the **feasibility of real-world implementation**, expressing uncertainty about how the dashboard could be integrated into existing clinical information systems (CIS): *“There are 60 to 70 different CIS (clinical information systems)*,* and you need access to all of them…”* (P10). Finally, three participants (3/14) suggested that to achieve broader adoption, the dashboard must be **extended beyond the SGED score** to include data on comorbidities, prediabetic patients, or other chronic conditions. Without such an expansion, they feared its usefulness would remain limited to a narrow segment of T2D management.

## Discussion

This study identified functional requirements (RQ1) and evaluated a high-fidelity prototype (RQ2) for a digital SGED score dashboard for T2D management in Swiss primary care. The requirement elicitation (RQ1) revealed broad consensus on core features, including reminder mechanisms for missing or overdue SGED-relevant assessments, color-coded flagging of critical values such as low nephropathy screening rates, and long-term trend analyses of SGED indicators at the practice level. At the same time, participants surfaced additional needs such as individual patient views, for example allowing healthcare professionals to review SGED-relevant indicators for specific patients, checklists of recommended assessments, and comorbidity tracking that extend beyond the current population-level scope of the SGED score. The prototype evaluation (RQ2) yielded a distinctly mixed picture. Participants valued the dashboard’s clear and intuitive design, its diverse visualizations of SGED performance indicators at the practice level, and the benchmarking functionality, describing it as engaging and efficient. However, half of the participants (7/14) saw limited benefit for daily clinical workflows. They cited the population-level nature of the SGED score, which aggregates data across patient populations rather than supporting decision-making for individual patients, the absence of individual patient data, potential additional administrative burden, and uncertainty about integration with existing clinical information systems. This tension between the recognized conceptual value of certain dashboard features and their questioned practical relevance is consistent with broader challenges in clinical dashboard adoption [31] and the successful scaling of new digital health technologies [32], and warrants closer examination below.

The mixed reception of the prototype is consistent with patterns observed in the broader healthcare dashboard literature. A recent scoping review of 118 dashboards found that only half involved end users in the dashboards design process, and that qualitative evaluation remains rare, with only 5% of studies relying exclusively on qualitative methods [31]. The iterative, user-centered approach adopted in this study thus represents a methodological strength, and the candid mixed feedback it elicited is itself a valuable finding that most dashboard projects never capture. Importantly, the limited perceived relevance for daily clinical workflows can be understood through the distinction between population-health management tools and point-of-care clinical decision support [33]. The SGED score dashboard is designed as a practice-level quality monitoring tool. It functions as an audit and feedback instrument rather than a system that supports individual patient decisions during consultations. Research on audit and feedback has shown that such interventions are more effective when they include specific targets and actionable recommendations [34]. The absence of such actionable elements in the current prototype may partly explain why participants questioned its practical utility. The functional requirements identified in this study, such as trend visualization, alert mechanisms, and benchmarking, are broadly consistent with those reported in recent clinical dashboard design frameworks [35] and systematic reviews of dashboard requirements [17]. This convergence suggests that the needs expressed by Swiss T2D healthcare professionals are not idiosyncratic but reflect common priorities in clinical dashboard design.

### Key challenges

One of the most critical and currently unresolved challenges in T2D management in Switzerland is the fragmented landscape of CIS [36]. This fragmentation poses a fundamental barrier to the development and implementation of interoperable systems [37, 38]. Moreover, the lack of interoperability leads to additional costs, as data often has to be entered or reconciled manually [39, 40]. Across interviews, it became evident that the absence of standardized interfaces or a commonly used national decision-support CIS is a core impediment. Each CIS operates in isolation today, meaning data cannot be easily exchanged or integrated across systems, making it difficult to compile and visualize the SGED score in a consistent and structured manner across multiple systems. In contrast to studies conducted in more standardized hospital environments, our participants emphasized the heterogeneity of CIS vendors and configurations in primary care, suggesting that interoperability challenges may be even more pronounced in this setting than previously described [17, 37]. This prevents automation, limits decision functionalities, and undermines the utility of performance feedback, and this is not only happening in the context of T2D [37]. Therefore, the challenge of bringing together disparate data must be considered both a technical prerequisite and the most urgent priority for implementing any kind of clinical dashboard. Without addressing this foundational issue, digital support systems such as the SGED visualization dashboard risk remaining conceptual rather than operational.

Another key challenge concerns the limited relevance of the SGED score dashboard in everyday clinical practice with respect to individual patients. While the SGED score is calculated based on aggregated patient data, the SGED score is a performance measure for the entire T2D population at a given practice [7]. Hence, visualizations of the SGED score serve as aggregated population-level indicators and not for individual patient details. Nonetheless, healthcare professionals rely on patient-specific data and tools tailored to clinical decision-making during consultations with T2D patients [17]. Prior work on diabetes dashboards and quality indicators has similarly shown that aggregated metrics are valued for audit and feedback, but are rarely used directly at the point of care [17, 19]. Our findings extend this evidence by showing that, in the context of the SGED score, clinicians explicitly distinguish between population-level and patient-level functionalities and consider the latter indispensable for daily practice. As a result, a visualization dashboard is more likely to only be used periodically for performance monitoring and quality assurance [19] rather than in day-to-day clinical routines. This population-oriented nature limits its perceived practicality for a broad range of healthcare professionals.

### Potential strategies for adoption

To increase the likelihood of successful adoption of a digital SGED dashboard, several implementation strategies can be considered. For the SGED score dashboard to be used at scale, it must be embedded into the daily workflows of primary care in a way that minimizes administrative burden while maximizing clinical usefulness. Interoperability is the central requirement for this [17, 41]. Consistent with previous implementation studies [17, 19], our findings suggest that usability alone is insufficient to drive adoption, structural integration into existing systems and workflows appears to be decisive. Data entered by patients or healthcare professionals must be synchronized seamlessly within the GP’s CIS. Such interoperability ensures that information is not siloed and can be accessed easily across different platforms. Technically, such interoperability could be facilitated through standardized application programming interfaces (APIs), such as those based on the HL7 FHIR standard, which would enable automated data extraction from heterogeneous CIS.

Two potential implementation pathways can be distinguished. First, a digital SGED score dashboard could function as a stand-alone secondary system, which would require standardized national interfaces to enable data exchange across different CIS. This approach would allow practices to maintain their existing software but would depend heavily on robust data integration standards, similar to modular, app-based approaches discussed in national interoperability initiatives [36, 37, 39]. Second, the dashboard could be integrated into existing decision-support systems or CIS. This would likely facilitate adoption, as healthcare professionals would not need to adapt to an entirely new system. Leveraging existing infrastructures could reduce technical complexity and would ensure a smoother workflow integration [42]. Nonetheless, an existing system would need to be open to integrating the identified functional requirements into their functionality. Whichever path is chosen, the emphasis must remain on reducing redundancy and additional workload to minimize further tasks for already pressured healthcare professionals [3, 4]. The dashboard should ideally provide automated data synchronization and actionable insights [17] rather than becoming another layer of administrative effort. Ensuring that the tool supports rather than disrupts existing practices will be decisive for its long-term acceptance.

In addition to these technical considerations, the dashboard’s practical relevance could be strengthened in two ways to increase its adoption likelihood. First, its value proposition should be framed around concrete, actionable use cases rather than descriptive score reporting alone. For example, identifying underperforming SGED indicators could prompt targeted interventions such as intensifying screening for T2D complications such as retinopathy or nephropathy. Long-term trend analysis could enable early detection of deteriorating care quality, and anonymized benchmarking could motivate practices to learn from higher-performing peers. Research on audit and feedback confirms that such interventions are more effective when they include specific targets and action plans [34]. Second, the intended user base should be reconsidered. Given the population-level focus of the SGED score, the dashboard may be particularly valuable for clinic administrators, quality managers, and managed care organizations, who have a more direct mandate for periodic quality monitoring and reporting than frontline clinicians engaged in individual patient care [33]. Positioning the dashboard as a quality management tool rather than a clinical workflow tool could better align its design with the needs of its most likely users.

### Implications for dashboard design

Based on our findings and in line with prior reviews of dashboard design in healthcare [35, 43], we can derive several practical recommendations for engineers and developers more broadly. First, interoperability with existing clinical information systems should be treated as a prerequisite rather than an afterthought. Second, the intended user and their decision context should be clearly defined early in the design process, as dashboards that attempt to serve multiple audiences with a single view risk failing all of them. Third, iterative user involvement throughout the design process is essential. Our study demonstrates that even with such involvement, critical mismatches between tool design and user expectations can emerge, underscoring the value of qualitative evaluation at an early prototype stage. Fourth, population-level quality dashboards should provide not only descriptive score visualizations but also actionable recommendations to maximize their practical utility.

### Future work

Several open questions remain regarding the feasibility and future of the SGED score dashboard. A central question is whether the system can be seamlessly integrated into current clinical workflows, using one of the potential implementation strategies from subsection 4.2. Another open issue is the rigidity of the SGED score itself [8]. Many interviewees noted that the score, in its current form, is too inflexible, lacking the customization needed to reflect diverse realities of T2D management, such as accounting for individual patient comorbidities or lifestyle factors. This rigidity reduces its direct applicability and limits healthcare professionals’ motivation to engage with it beyond the financial incentives received as compensation, echoing broader critiques of fixed diabetes quality metrics that insufficiently capture multimorbidity and self-management demands [8, 19]. Therefore, future work should explore whether the SGED score could be made more adaptable to the needs of individual practices, potentially by turning it into a more personalized and individual score. For example, by allowing customizable indicators or integrating additional chronic disease metrics [8, 17]. Finally, there remains a broader strategic and policy-level question: how can interoperability and data sharing be promoted at a national level to enable digital quality monitoring tools like the SGED score dashboard? Addressing these systemic issues would not only enhance the feasibility of this prototype but also contribute to the broader digital transformation of chronic care in Switzerland.

## Limitations

This study has several limitations that should be acknowledged. First, the sample size of 14 participants (derived from four group interviews and six individual interviews) was relatively small, which limits the generalizability of the findings. Furthermore, participants were not sampled representatively across Switzerland, since the interviewees were from the German-speaking region. Second, there may have been a selection bias, as those with a particular interest in digital health or structured diabetes care may have been more likely to participate, potentially skewing the feedback. Moreover, despite being conducted online, the interviews may have been subject to social desirability bias, with participants expressing more overly positive responses toward the prototype than in a more neutral setting. Third, the study examined a hypothetical SGED score dashboard rather than an operational system. Consequently, many of the insights reflect anticipated behavior rather than observed usage. While participants provided thoughtful reflections on both positive and negative aspects of the prototype, their responses may not fully predict actual adoption or engagement under real-world conditions, especially when considering variations in workload, clinical routines, or policy constraints. Fourth, the transcripts were only analyzed by one researcher, which may have introduced subjective bias and limited the reliability of the interpretations. Finally, while this study focused on identifying functional requirements and generating an initial prototype for a visualization dashboard for the SGED score, it did not investigate practical aspects of implementation, such as potential commercial partners or explicit integration pathways within existing systems. Future work will need to address potential integration pathways to determine the feasibility, effectiveness, and sustainability of the dashboard in clinical practice.

## Conclusion

This study examined the design and development of a prototype of a potential SGED score dashboard, a tool intended to provide an aggregated overview of T2D care quality at the practice level in Switzerland. Through qualitative interviews with key stakeholders, including GPs, MPAs, and MPCs, we identified key functional requirements, user preferences, and barriers to implementation. These challenges included system fragmentation, workflow integration challenges, and the population-level focus of the SGED score. While participants generally recognized the dashboard’s potential to support performance monitoring and quality improvement, successful adoption will ultimately depend on interoperability, usability, and alignment with existing clinical routines. Taken together, the identified functional requirements (RQ1) and user prototype evaluation (RQ2) can inform the further development and evaluation of digital tools that enhance chronic disease management and inform strategies for scalable implementation in primary care.

## Supplementary Information

Below is the link to the electronic supplementary material.

Supplementary Material 1

Supplementary Material 2

Supplementary Material 3

Supplementary Material 4

Supplementary Material 5

Supplementary Material 6

Supplementary Material 7

Supplementary Material 8

Supplementary Material 9

## Data Availability

All data generated or analyzed during this study are included in this article and its supplementary information files (Additional Files 1-9).

## Abbreviations

T2D: Diabetes mellitus type 2
SGED: Swiss Society of Endocrinology and Diabetology
GP: General Practitioner
MPA: Medical Practice Assistant
MPC: Medical Practice Coordinator
MoSCoW: “Must Have”, “Should Have”, “Could Have”, “Won’t Have”
COREQ: Consolidated Criteria for Reporting Qualitative Research
CIS: Clinical Information System
