# Supplementary material for: A care quality dashboard for general practitioners managing patients with diabetes mellitus type 2: user-centered design and prototype evaluation
Source: BMC Med Inform Decis Mak. 2026 May 9;26:234. doi: 10.1186/s12911-026-03492-3 (PMC13326401; doi:10.1186/s12911-026-03492-3)
Supplement: Supplementary file 1 — Supplementary Material 1 [file 12911_2026_3492_MOESM1_ESM.docx]

Coding Framework

Table 1. Clinical Workflow and Knowledge

| **Themes** | **Codes** |
| --- | --- |
| SGED Score Awareness & Utilization | - Knowledge about SGED score - Active use of SGED score in practice - Adapted or partial use of SGED score - Lack of SGED score usage |
| Professional Role & Experience | - Professional role (GP / MPK / MPA / Endocrinologist) - Years of experience (3–5 y, 5–10 y, +10 y) - Number of patients per week (0–25, 25–50, 75–100) |
| Clinical Workflow Practices | - Use of structured tools (checklists, protocols, …) - Lifestyle counseling and monitoring - Population-level vs. individual patient management |

Table 2. Functional Requirements and Data Management

| **Theme** | **Codes** |
| --- | --- |
| Data Visualization | - Demographic overview over the T2D collective - Long-term trends - Variety of visualizations - Labeling and flagging of critical values - Reminder and alert mechanism - Filtering options for subpopulations - Visualizations of the overall SGED score |
| Benchmarking & Performance Comparison | - Benchmarking within the network - Anonymized benchmarking - Benchmarking promotes transparency - Perceived usefulness of benchmarking - Export or reporting function |
| Additional Functional Needs | - laboratory data - View individual patients - Tracking individual goals - AI or decision support for insights - Patient comorbidities |

Table 3. Feedback on Prototype and User Experience

| **Theme** | **Codes** |
| --- | --- |
| User Interface & Accessibility | - Positive feedback regarding prototype - Clear, straightforward user interface - Customizable user flow - Role-based access - Suggestions for improvement - Difficulties with interpretation of data - Limited flexibility and lack of interactivity |
| Patient Engagement Features | - Individual patient overviews - Active patient participation - Monitoring lifestyle & behavior - Inclusion of pre-diabetics |

Table 4. Digitalization and System Integration Challenges

| **Theme** | **Codes** |
| --- | --- |
| Systemic & Structural Barriers | - Administrative workload - Financial or resource constraints |
| Integration & Interoperability | - Interfaces with other systems - Avoidance of secondary tools - Cloud-based solutions for shared access |
| Adoption & Acceptance Challenges | - Limited perceived benefit - Feasibility of real-world implementation - Difficulty integrating new workflows into practice |
