# Supplementary material for: A care quality dashboard for general practitioners managing patients with diabetes mellitus type 2: user-centered design and prototype evaluation
Source: BMC Med Inform Decis Mak. 2026 May 9;26:234. doi: 10.1186/s12911-026-03492-3 (PMC13326401; doi:10.1186/s12911-026-03492-3)
Supplement: Supplementary file 2 — Supplementary Material 2 [file 12911_2026_3492_MOESM2_ESM.docx]

Semi-structured interview guide

Introduction and background:

- Could you please briefly introduce yourself? What is your name, what is your role in caring for patients with type 2 diabetes, and how long have you been doing this work?
- Approximately how many type 2 diabetes patients do you care for in your practice?
- Do you actively use the SGED score in your daily work? Do you use it as a performance indicator, or do you see the score more as a basis/guideline?

Input 1 Brainstorming for functional requirements (new ideas):

In the first part of the interview, we would like to jointly develop functional requirements for the SGED score dashboard. Feel free to start by brainstorming: What requirements or functions come to mind when you think of a clinical population dashboard for the SGED score?

- What information about the SGED score would be particularly helpful to you in your daily work?
- What actions would you ideally like to be able to take after reviewing the SGED score?
- Which key performance indicators (KPIs) or visualizations related to the SGED score would be particularly helpful to you?

How would you prefer to see the score, or its individual components displayed (e.g., tables, bar charts, traffic light system)?

Input 2: Feedback on existing functional requirements from P1 (validation):

Based on a literature review and the first round of interviews during our initial project, we were able to identify some initial functional requirements. I will now present these to you. What is your assessment of the proposals so far?

Is there anything missing from the current list?

Input 3: Prioritization of all identified functional requirements (MoSCoW framework):

As a next step, I would like to prioritize the identified requirements and functionalities. This will ensure that all important features are included in our final prototypes. We will use the MoSCoW framework for this, which distinguishes between "must-have", "should-have", "could-have", and "won't-have".

Input 4: Feedback on the low-fidelity prototype:

To conclude the interview, we would like your feedback on our current low-fidelity prototype. This prototype serves as the first visual representation of the requirements gathered so far and demonstrates how the SGED Score dashboard could be structured. We are particularly interested in your perception of the user flow, the presentation of functions, and the layout, as well as any areas where you see potential for improvement. Based on your feedback, the MoSCoW prioritization, and the new functional requirements identified during this interview, we will then further refine and optimize the prototype.

**Perception & Comprehensibility**

- What is your first impression of the dashboard's layout and structure?
- Can you visualize how you would navigate through the dashboard?

**Presentation of Functionalities & Design**

- Which elements of the prototype immediately catch your eye?
- Is the information presented (e.g., score values, charts, tables) understandable to you?
- How do you find the presentation of the SGED score in the population overview? Is the information visualized clearly and understandably?
- Do you feel supported or rather confused by the layout and structure?

**Suggestions for Improvement & Prioritization**

- If you could change one thing about this prototype immediately, what would it be and why?
- Are there any features that are currently missing from the prototype?
- Are there any features that you think are superfluous or presented in an overly complicated way in the prototype?

**Final Reflection**

Could you imagine using this dashboard in your daily work? What would help you or what would be missing?

**German Version**

Einführung und Hintergrund:

- Könnten Sie sich bitte kurz vorstellen? Wie ist Ihr Name, in welcher Funktion betreuen Sie Patientinnen und Patienten mit T2D, und wie lange üben Sie diese Tätigkeit bereits aus?
- Wie viele T2D Patienten betreuen Sie ungefähr in Ihrer Praxis?

Verwenden Sie den SGED Score aktiv in Ihrem Arbeitsalltag? Nutzen Sie ihn als Performance-Indikator oder sehen Sie den Score eher als Grundlage/ Orientierungshilfe?
Input 1 Brainstorming für funktionale Anforderungen (neue Ideen):

Im ersten Teil des Interviews möchten wir gemeinsam funktionale Anforderungen für das SGED-Score-Dashboard erarbeiten. Sie können gerne einfach mit dem Brainstorming beginnen: Welche Anforderungen oder Funktionen kommen Ihnen in den Sinn, wenn Sie an ein klinisches Populationsdashboard für den SGED Score denken?

- Welche Informationen zum SGED Score wären für Sie im Berufsalltag besonders hilfreich?
- Welche Handlungen möchten Sie idealerweise nach dem Betrachten des SGED Scores durchführen können?
- Welche Kennzahlen oder Visualisierungen wären für Sie im Zusammenhang mit dem SGED Score besonders hilfreich?

Auf welche Weise würden Sie sich den Score oder dessen Einzelkomponenten am liebsten anzeigen lassen (z.B. Tabellen, Balkendiagramme, Ampelsystem)?

Input 2: Feedback zu bestehenden funktionalen Anforderungen aus P1 (Validierung):

Aus einer Literaturrecherche sowie der ersten Interviewrunde im Rahmen unseres ersten Projekts konnten wir bereits erste funktionale Anforderungen identifizieren. Diese werde ich Ihnen nun vorstellen. Wie beurteilen Sie die bisherigen Vorschläge?

Fehlt Ihnen etwas in der bisherigen Liste?

Input 3: Priorisierung aller identifizierten funktionalen Anforderungen (MoSCoW-Framework):

In einem nächsten Schritt würde ich gerne die identifizierten Anforderungen und Funktionalitäten priorisieren. Dadurch können wir sicherstellen, dass alle wichtigen Features in unseren endgültigen Prototypen enthalten sind. Dafür werden wir das MoSCoW Framework verwenden, bei dem zwischen „Must-have“, „Should-have“, „Could-have“ und „Won’t-have“ unterschieden wird.

Input 4: Feedback zum low-fidelity prototype:

Zum Abschluss des Interviews möchten wir gerne Ihre Rückmeldung zu unserem aktuellen Low-Fidelity-Prototypen erhalten. Dieser Prototyp dient als erste visuelle Umsetzung der bisher gesammelten Anforderungen und zeigt, wie das SGED Score-Dashboard grundsätzlich aufgebaut sein könnte. Uns interessiert dabei besonders, wie Sie den Nutzerfluss, die Darstellung der Funktionen und das Layout wahrnehmen, und wo Sie noch Verbesserungspotenzial sehen. Basierend auf Ihrem Feedback, der MoSCoW-Priorisierung sowie den neuen funktionalen Anforderungen aus diesem Interview werden wir den Prototyp anschließend weiter anpassen und optimieren.

**Wahrnehmung & Verständlichkeit**

- Was ist Ihr erster Eindruck vom Aufbau und der Struktur des Dashboards?
- Können Sie nachvollziehen, wie Sie sich durch das Dashboard bewegen würden?

**Darstellung der Funktionalitäten & Design**

- Welche Elemente des Prototyps springen Ihnen sofort ins Auge?
- Sind die dargestellten Informationen (z. B. Score-Werte, Diagramme, Tabellen) für Sie verständlich?
- Wie empfinden Sie die Darstellung des SGED Scores in der Populationsübersicht? Werden die Informationen für Sie klar und verständlich visualisiert?
- Fühlen Sie sich durch das Layout und die Struktur unterstützt oder eher verwirrt?

**Verbesserungsvorschläge & Priorisierung**

- Wenn Sie eine Sache an diesem Prototyp sofort ändern könnten, was wäre das und warum?
- Gibt es aus Ihrer Sicht Funktionen, die im Prototyp zurzeit noch fehlen?
- Gibt es aus Ihrer Sicht Funktionen, die im Prototyp überflüssig oder zu kompliziert dargestellt werden?

**Abschließende Reflexion**

Könnten Sie sich vorstellen, dieses Dashboard in Ihrem Arbeitsalltag zu nutzen? Was würde Ihnen dabei helfen oder fehlen?
