# Supplementary material for: A care quality dashboard for general practitioners managing patients with diabetes mellitus type 2: user-centered design and prototype evaluation
Source: BMC Med Inform Decis Mak. 2026 May 9;26:234. doi: 10.1186/s12911-026-03492-3 (PMC13326401; doi:10.1186/s12911-026-03492-3)
Supplement: Supplementary file 3 — Supplementary Material 3 [file 12911_2026_3492_MOESM3_ESM.docx]

Study information and consent form

**Study Title: Digitalization of Diabetes Management Score (D-SGED-S) – Project 2**

Dear Participant,

You are invited to participate in a research study on the digitalization of the Diabetes Management Score (D-SGED-S). This study information sheet provides details about the nature and methods of the study in which you will participate. It is therefore important that you read the following information carefully.

The goal of the project is to digitalize the Diabetes Management Score of the Swiss Society of Endocrinology and Diabetology (SGED), leading to the development of the Digital SGED Score (D-SGED-S). This score is intended to support general practitioners (GPs) in making informed decisions and improving the treatment of type 2 diabetes (T2D). As part of this study, an initial prototype will be developed, and the optimal way to visualize the SGED Score in a clinical population dashboard will be investigated. Your participation in this group interview will help us better understand the needs and expectations regarding the D-SGED-S prototype.

Please note that no personal or sensitive data that could be used to identify you will be collected. Your participation remains anonymous, and you can withdraw from the study at any time. This study contains no hidden information and no deception.

Study Procedure:

You are invited to participate in a group interview as part of this project. Together with other healthcare professionals from your practice who care for patients with type 2 diabetes (GP, medical practice assistant, medical practice nurse, advanced practice nurse, etc.), you will participate in this group interview. The interview will focus on your experiences, expectations, and opinions regarding the digitization of the SGED score. The focus is on a human-centered design approach with iterative development cycles to create a user-friendly and practical dashboard for the SGED population.

The group interview will be conducted as an online meeting via MS Teams, and the access information (meeting link and, if applicable, password) will be sent to you in advance via email. Your participation will take approximately 60 minutes. Please note that the duration may vary, as you will be participating in an interactive study.

Voluntary Participation:

You may withdraw your consent at any time. This is possible without giving reasons. If you withdraw your consent, all forms you have completed will be destroyed immediately and will not be used for the study. Withdrawing your consent has no consequences, and you will not receive any financial compensation for participating in the study.

Recording:

Because the study team will subsequently transcribe and analyze the interviews conducted as part of this study in anonymized form, the interview will be recorded. The recording serves solely for the purpose of subsequent transcription and will be completely deleted afterward.

Confidentiality of Study Results:

All measures taken within this study are solely for scientific purposes. Apart from your gender, age, and information about your professional experience, no sensitive or personal data will be collected. All information gathered within this study is confidential, and appropriate data storage is used to ensure that only members of the research team have access to the interviews.

Your personal data (name, email address) will be stored securely and separately from your coded responses for 90 days after the study is completed. You can contact us during this period to have your data removed from our database. After this period, your personal data will be permanently deleted.

The coded responses (no personal data) may be made publicly available via the Open Science Framework (OSF) if scientific reports from the current study are published in peer-reviewed journals and/or conference presentations.

Research Group:

In close collaboration with Prof. Dr. med. Michael Brändle, Prof. Dr. Tobias Kowatsch (tobias.kowatsch@unisg.ch) and Lola Jo Ackermann (lolajo.ackermann@student.unisg.ch) are coordinating this study. They can be contacted with any questions or comments. This study has received approval for exemption from formal review by the Ethics Committee of the University of St. Gallen. Complaints regarding the study can be addressed to Prof. Dr. Tobias Kowatsch.

Informed Consent:

I hereby declare that: I have read and understood the information sheet for the study. I had the opportunity to ask clarifying questions. My questions were answered appropriately. I had sufficient time to decide whether or not I wish to participate in this study.

I am aware that participation in this study is entirely voluntary. I understand that I can withdraw my consent at any time during the study. I do not have to provide a reason.

I agree that my anonymized data may be used for the purposes stated in the information sheet. I understand that I can only participate in the study by signing the consent form.

Name (participant) Name (researcher)

Date Date

Signature Signature

**German Version**

**Studientitel: Digitalization of Diabetes Management Score (D-SGED-S) – Project 2**

Sehr geehrte Teilnehmerin, sehr geehrter Teilnehmer,

sie sind eingeladen, an einer Forschungsstudie zur Digitalisierung des Diabetes

Management Scores (D-SGED-S) teilzunehmen. Dieses Studieninformationsblatt enthält Einzelheiten über die Art und die Methoden der Studie, an der Sie teilnehmen werden. Es ist daher wichtig, dass Sie die folgenden Informationen sorgfältig lesen.

Das Ziel des Projekts ist die Digitalisierung des Diabetes Management Scores der Schweizerischen Gesellschaft für Endokrinologie und Diabetologie (SGED), was zur Entwicklung des Digitalen SGED-Scores (D-SGED-S) führen soll. Dieser Score soll GPs dabei unterstützen, fundierte Entscheidungen zu treffen und die Behandlung von T2D zu verbessern. Im Rahmen dieser Studie wird ein erster Prototyp entwickelt und untersucht, wie der SGED-Score optimal in einem klinischen Populationsdashboard visualisiert werden kann. Ihre Teilnahme an diesem Gruppeninterview wird uns helfen, die Bedürfnisse und Erwartungen bezüglich des D-SGED-S Prototyps besser zu verstehen.

Bitte beachten Sie, dass keine persönlichen oder sensiblen Daten gesammelt werden, die zur Identifizierung Ihrer Person verwendet werden können. Ihre Teilnahme bleibt anonym und Sie können jederzeit von der Studie zurücktreten. Diese Studie enthält keine versteckten Informationen und keine Täuschungen

Studienablauf:

Sie sind eingeladen, an einem Gruppeninterview im Rahmen dieses Projekts teilzunehmen. Gemeinsam mit weiteren Diabetes-Typ-2 betreuenden Fachpersonen aus ihrer Praxis (GP, MPA, MPK, APN, etc.) werden sie an diesem Gruppeninterview teilnehmen. Das Interview wird sich auf Ihre Erfahrungen, Erwartungen und Meinungen zur Digitalisierung des SGED-Scores konzentrieren. Im Fokus steht ein Human-Centered Design-Ansatz mit iterativen Entwicklungszyklen, um ein benutzerfreundliches und praxisnahes Dashboard für die SGED-Population zu gestalten.

Das Gruppeninterview wird im Rahmen eines Onlinemeetings via MS Teams durchgeführt und die Zugangsdaten (Meeting-Link und allenfalls Passwort) werden im Vorfeld via E-Mail zugesendet. Die Gesamtdauer Ihrer Teilnahme beträgt ungefähr 60 Minuten. Bitte beachten Sie, dass die Dauer variieren kann, da Sie an einer interaktiven Studie teilnehmen.

Freiwillige Teilnahme:

Sie können Ihre Einwilligung jederzeit widerrufen. Dies ist ohne Angabe von Gründen möglich. Wenn Sie Ihre Einwilligung widerrufen, werden alle von Ihnen ausgefüllten Formulare umgehend vernichtet und nicht für die Studie verwendet. Der Widerruf der Einwilligung hat keine Konsequenzen und Sie erhalten keine finanzielle Entschädigung für die Teilnahme an der Studie.

Aufzeichnung:

Da das Studienteam, die im Rahmen dieser Studie geführten Interviews im Nachgang transkribiert und in anonymisierter Form auswertet, wird das Interview aufgezeichnet. Die Aufzeichnung dient hierbei ausschliesslich der nachträglichen Transkription und wird im Anschluss vollständig gelöscht.

Vertraulichkeit der Studienergebnisse:

Alle Massnahmen im Rahmen dieser Studie dienen ausschliesslich wissenschaftlichen Zwecken. Ausser Ihrem Geschlecht, Ihrem Alter und Daten zu Ihrer Berufserfahrung werden keine sensiblen oder persönlichen Daten erfasst. Alle im Rahmen dieser Studie gesammelten Informationen sind vertraulich und es wird eine entsprechende Datenspeicherung verwendet, um sicherzustellen, dass nur Mitglieder des Forschungsteams Zugriff auf die Interviews haben.

Ihre persönlichen Daten (Name, E-Mail-Adresse) werden nach Abschluss der Studie 90 Tage lang sicher und getrennt von Ihren codierten Antworten aufbewahrt. Sie können uns während dieser Zeit kontaktieren, um Ihre Daten aus unserer Datenbank zu entfernen. Nach diesem Zeitraum werden Ihre persönlichen Daten endgültig gelöscht.

Die codierten Antworten (keine persönlichen Daten) können über das OSF (Open Science Framework) öffentlich zugänglich gemacht werden, wenn wissenschaftliche Berichte aus der aktuellen Studie in von Experten begutachteten Zeitschriften und/oder Konferenzpräsentationen veröffentlicht werden.

Forschungsgruppe:

In enger Zusammenarbeit mit **Prof. Dr. med. Michael Brändle** koordinieren Prof. Dr. Tobias Kowatsch ([tobias.kowatsch@unisg.ch](mailto:tobias.kowatsch@unisg.ch)) und Lola Jo Ackermann

([lolajo.ackermann@student.unisg.ch](mailto:lolajo.ackermann@student.unisg.ch)) diese Studie. Sie können für Fragen oder Kommentare kontaktiert werden. Diese Studie erhielt eine Genehmigung zur Befreiung von einer formellen Überprüfung durch die Ethikkommission der Universität St. Gallen. Beschwerden über die Studie können an Prof. Dr. Tobias Kowatsch gerichtet werden.

Einverständniserklärung:

Hiermit erkläre ich, dass:

Ich das Informationsblatt zur Studie gelesen und verstanden habe. Ich hatte die Möglichkeit, Fragen zur Klärung zu stellen. Meine Fragen wurden angemessen beantwortet. Ich hatte ausreichend Zeit, um zu entscheiden, ob ich an dieser Studie teilnehmen möchte oder nicht.

Mir ist bewusst, dass die Teilnahme an dieser Studie völlig freiwillig ist. Ich weiss, dass ich meine Einwilligung jederzeit während der Studie widerrufen kann. Ich muss dafür keinen Grund angeben.

Ich bin damit einverstanden, dass meine verschlüsselten Daten für die im Informationsblatt genannten Zwecke verwendet werden. Ich verstehe, dass ich nur mit Unterzeichnung der Einverständniserklärung an der Studie teilnehmen kann.

Name (Teilnehmer/in) Name (Forscher)

Datum Datum

Unterschrift Unterschrift
