# Supplementary material for: A care quality dashboard for general practitioners managing patients with diabetes mellitus type 2: user-centered design and prototype evaluation
Source: BMC Med Inform Decis Mak. 2026 May 9;26:234. doi: 10.1186/s12911-026-03492-3 (PMC13326401; doi:10.1186/s12911-026-03492-3)
Supplement: Supplementary file 4 — Supplementary Material 4 [file 12911_2026_3492_MOESM4_ESM.docx]

Consolidated criteria for reporting qualitative studies using the COREQ (Consolidated Criteria for Reporting Qualitative Research) 32-item checklist

| **No** | **Item Guide** | **Questions/Descriptions** | **Reported page?** |
| --- | --- | --- | --- |
| **Domain 1: Research team and reflexivity** | | | |
| Personal Characteristics | | | |
| 1 | Interviewer/facilitator | Which author/s conducted the interview or  focus group? | Page 1 |
| 2 | Credentials | What were the researcher’s credentials? E.g. PhD, MD | n/a |
| 3 | Occupation | What was their occupation at the time of the study? | Page 1 |
| 4 | Gender | Was the researcher male or female? | n/a |
| 5 | Experience and training | What experience or training did the researcher have? | Page 20 |
| Relationship with participants | | | |
| 6 | Relationship established | Was a relationship established prior to study commencement? | n/a |
| 7 | Participant knowledge of the Interviewer | What did the participants know about the researcher? *e.g. personal goals, reasons for doing the research* | n/a |
| 8 | Interviewer characteristics | What characteristics were reported about the Interviewer/ facilitator? *e.g. Bias, assumptions, reasons and interests in the research topic* | Page 20 |
| **Domain 2: study design** | | | |
| Theoretical framework | | | |
| 9 | Methodological orientation and Theory | What methodological orientation was stated to underpin the study? *e.g. grounded theory, discourse analysis, ethnography, phenomenology, content analysis* | Page 4 |
| Participant selection | | | |
| 10 | Sampling | How were participants selected? *e.g. purposive, convenience, consecutive, snowball* | Page 7 |
| 11 | Method of approach | How were participants approached? *e.g. face-to-face, telephone, mail, email* | n/a |
| 12 | Sample size | How many participants were in the study? | Page 4 |
| 13 | Non-participation Setting | How many people refused to participate or dropped out? Reasons? | n/a |
| Setting | | | |
| 14 | Setting of data collection | Where was the data collected? *e.g. home, clinic, workplace* | Page 7 |
| 15 | Presence of non-participants | Was anyone else present besides the participants and researchers? | n/a |
| 16 | Description of sample | What are the important characteristics of the sample? *e.g. demographic data, date* | Additional File 4 |
| Data Collection | | | |
| 17 | Interview guide | Were questions, prompts, guides provided by the authors? Was it pilot tested? | Additional File 3 |
| 18 | Repeat interviews | Were repeat interviews carried out? If yes, how many? | n/a |
| 19 | Audio/visual recording | Did the research use audio or visual recording to collect the data? | Page 7 |
| 20 | Field notes | Were field notes made during and/or after the interview or focus group? | Page 7 |
| 21 | Duration | What was the duration of the interviews or focus group? | Additional File 4 |
| 22 | Data saturation | Was data saturation discussed? | Page 7 |
| 23 | Transcriptions returned | Were transcripts returned to participants for comment and/or correction? | n/a |
| **Domain 3: analysis and findings** | | | |
| Data analysis | | | |
| 24 | Number of data coders | How many data coders coded the data? | Page 19 |
| 25 | Description of the coding tree | Did authors provide a description of the coding tree? | Additional File 5 |
| 26 | Derivation of themes | Were themes identified in advance or derived from the data? | Page 7 |
| 27 | Software | What software, if applicable, was used to manage the data? | Page 7 |
| 28 | Participant checking | Did participants provide feedback on the findings? | n/a |
| Reporting | | | |
| 29 | Quotations presented | Were participant quotations presented to illustrate the themes / findings? Was each quotation identified? *e.g. participant number* | Pages 8 – 14 (results) |
| 30 | Data and findings consistent | Was there consistency between the data presented and the findings? | Page 16 |
| 31 | Clarity of major themes | Were major themes clearly presented in the findings? | Pages 8 – 14 (results) |
| 32 | Clarity of minor themes | Is there a description of diverse cases or discussion of minor themes? | Pages 8 – 14 (results) |
