# Supplementary material for: A care quality dashboard for general practitioners managing patients with diabetes mellitus type 2: user-centered design and prototype evaluation
Source: BMC Med Inform Decis Mak. 2026 May 9;26:234. doi: 10.1186/s12911-026-03492-3 (PMC13326401; doi:10.1186/s12911-026-03492-3)
Supplement: Supplementary file 5 — Supplementary Material 5 [file 12911_2026_3492_MOESM5_ESM.docx]

Predefined functional requirements for the dashboard

| **No** | **Feature** | **Description** |
| --- | --- | --- |
| 1 | Overview over the entire managed T2D population (1,2) | Provide information and statistics about all managed T2D patients, enabling population-level insights. |
| 2 | Labeling and flagging system (2–4) | Use color coding to visually highlight abnormal or critical values to draw attention to the matter. |
| 3 | Reminder and alert mechanism (2–4) | Show notifications about required follow-ups, overdue measurements, or significant changes in the SGED score. |
| 4 | Visualizations (2,3) | Provide graphical representations of key indicators for intuitive data interpretation. |
| 5 | Filtering options for subpopulations (3) | Enable filtering by specific criteria such as age, gender, or treatment duration to focus on relevant patient segments. |
| 6 | Export or reporting functions (3,4) | Allow users to export data or generate customized reports for documentation, analysis, or communication with stakeholders. |
| 7 | See trends over time (2,3) | Display data trends to assess the effectiveness of interventions and SGED score progress over time. |
| 8 | Benchmarking options (4) | Let users compare their SGED score against peer practices in their network. |
| 9 | Role-based access for staff (4) | Assign different access levels and permissions to team members according to their role *(e.g., physician, nurse, administrator)*. |
| 10 | Configuration possibilities for user workflow & individual preferences (3,4) | Support customization of workflows, and notification settings to match user roles and preferences. |
| 11 | Display information about the SGED score (2) | Show detailed information about the SGED score, including its components and calculation logic. |

1. Dagliati A, Sacchi L, Tibollo V, Cogni G, Teliti M, Martinez-Millana A, et al. A dashboard-based system for supporting diabetes care. J Am Med Inform Assoc JAMIA. 2018 Feb 2;25(5):538–47.

2. Giger OF, Ackermann LJ, Prinicpe M, Meier S, Fleisch E, Gallani S, et al. Advancing Primary Care for Type-2 Diabetes Management: Stakeholder Perspectives on Digital Quality Monitoring in Switzerland: A Qualitative Interview Study [Internet]. 2025. Available from: https://preprints.jmir.org/preprint/82960

3. 33-Funktions-Checkliste-fuer-Praxissoftware-d-v1.pdf [Internet]. [cited 2025 Oct 16]. Available from: https://www.equam.ch/wp-content/uploads/2024/10/33-Funktions-Checkliste-fuer-Praxissoftware-d-v1.pdf

4. Rabiei R, Almasi S. Requirements and challenges of hospital dashboards: a systematic literature review. BMC Med Inform Decis Mak. 2022 Nov 8;22(1):287.
