# Supplementary material for: A care quality dashboard for general practitioners managing patients with diabetes mellitus type 2: user-centered design and prototype evaluation
Source: BMC Med Inform Decis Mak. 2026 May 9;26:234. doi: 10.1186/s12911-026-03492-3 (PMC13326401; doi:10.1186/s12911-026-03492-3)
Supplement: Supplementary file 6 — Supplementary Material 6 [file 12911_2026_3492_MOESM6_ESM.docx]

Interviewee’s backgrounds

| Participant | Profession | Duration of Interview | Years of Experience | Canton |
| --- | --- | --- | --- | --- |
| B1 | GP | 55min | 5 | St. Gallen |
| B2 | MPA |  | 9 |  |
| B3 | GP/ Educator | 44min | 17 | St.Gallen |
| B4 | GP | 41min | 6 | St.Gallen |
| B5 | GP | 49min | 25 | St.Gallen |
| B6 | MPC |  | 11 |  |
| B7 | GP/ Advisor | 35min | 25 | St.Gallen |
| B8 | GP | 43min | 26 | Bern |
| B9 | MPC |  | 10 |  |
| B10 | Head of FIRE / Senior Physician | 49min | 12 | Zürich |
| B11 | Head of Research of FIRE / Specialist Physician |  | 13 |  |
| B12 | MPA | 43min | 6 | St.Gallen |
| B13 | Senior Endocrinology Physician | 29min | 15 | Luzern |
| B14 | MPC | 49min | 8 | Luzern |
