# Supplementary material for: A care quality dashboard for general practitioners managing patients with diabetes mellitus type 2: user-centered design and prototype evaluation
Source: BMC Med Inform Decis Mak. 2026 May 9;26:234. doi: 10.1186/s12911-026-03492-3 (PMC13326401; doi:10.1186/s12911-026-03492-3)
Supplement: Supplementary file 7 — Supplementary Material 7 [file 12911_2026_3492_MOESM7_ESM.docx]

Narrative elements for the prototype

Persona:

**
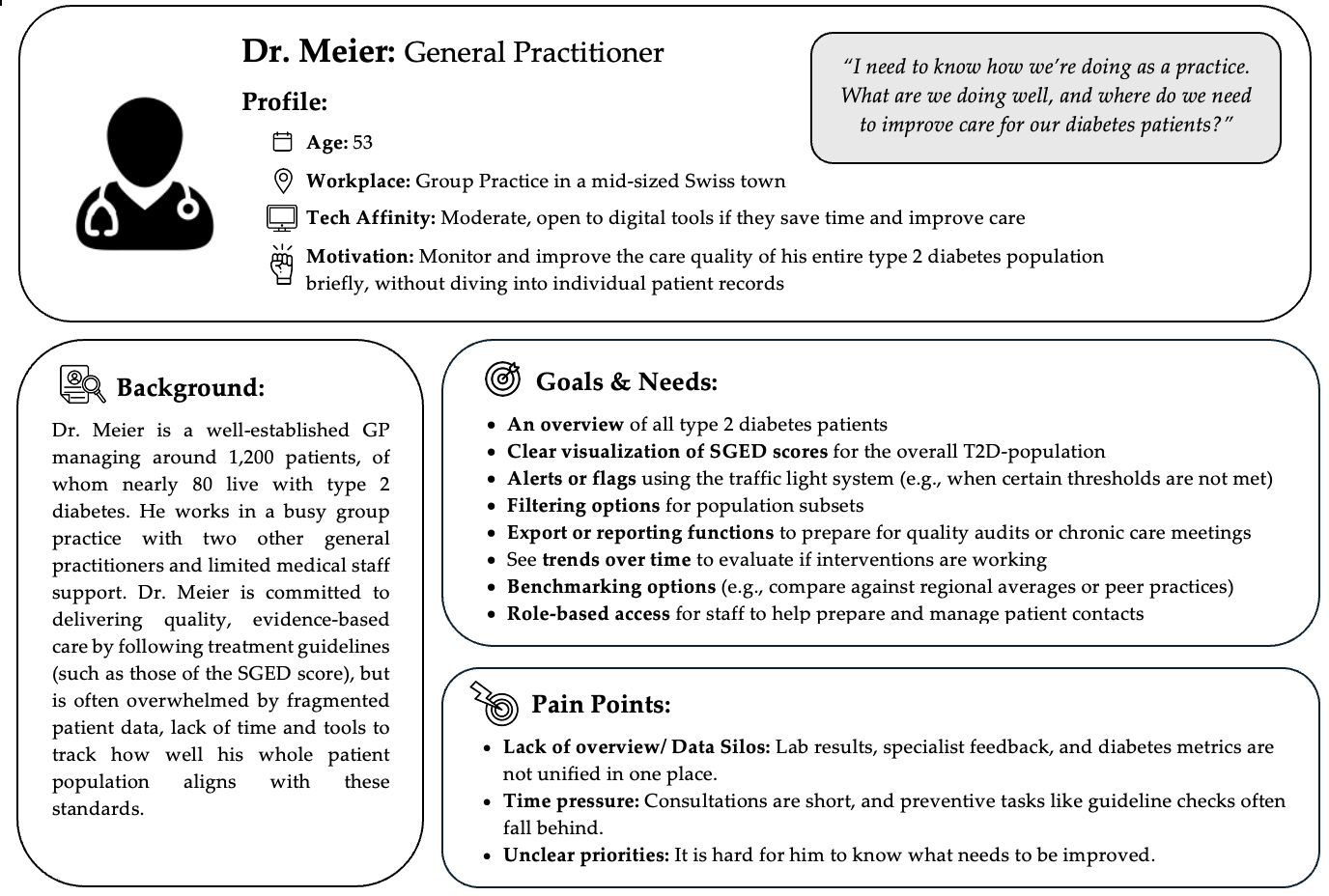
**

User Stories:

1. As a general practitioner, I want to see an overview of all my T2D patients in one dashboard including the demographics, so that I can understand their status. (Main population dashboard view)
2. As a general practitioner, I want the SGED score to be calculated and visualized automatically, so that I can assess the quality of care and identify areas for improvement. (Overall SGED score view)
3. As a general practitioner, I want critical metrics to be flagged and displayed using a coloring system, so that I can prioritize those. (Overall SGED score view)
4. As a general practitioner, I want to filter my patient population by certain criteria, so that I can focus on specific subgroups during chronic care planning. (Patient subgroup view)
5. As a general practitioner, I want to track trends in the SGED score over time, so that I can evaluate whether recent interventions are effective or where systemic issues arose in the past. (Trends view)
6. As a general practitioner, I want to compare my practice’s diabetes care performance to other practices in my network or benchmarks provided by health insurances, so that I can assess how we are performing relative to peers. (Benchmarking view)
7. As a general practitioner, I want to export the dashboard data into a report format, so that I can use it during team meetings or quality audits. (Report export view)
8. As a general practitioner, I want my medical staff to access the dashboard with role-specific views. (Login & role-based access view)
9. As a general practitioner, I want to customize the dashboard to my preferences, so that it fits into my daily workflow and time constraints. (Settings/ customizability view)
10. As a general practitioner, I want to view information about the SGED score and its values, so that I know how it is composed and how it is calculated. (SGED information view)

Scenario:

On Friday morning, Dr. Meier logs into the clinical dashboard and he sees a high-level overview of the practice’s T2D population, broken down by key metrics. He sees that only 64% of patients meet the HbA1c target, which is highlighted in light blue. This prompts him to look at trends: over the past 6 months, that number has dropped by 8%, coinciding with the departure of their diabetes nurse. He applies a filter to view patients over 70 years old and notes that only 32% in this group are meeting LDL cholesterol targets, a red indicator. He generates a population subgroup report, adds a benchmark comparison with 5 other practices from his network, and exports the findings for a later scheduled discussion with his team.
