# Supplementary material for: A care quality dashboard for general practitioners managing patients with diabetes mellitus type 2: user-centered design and prototype evaluation
Source: BMC Med Inform Decis Mak. 2026 May 9;26:234. doi: 10.1186/s12911-026-03492-3 (PMC13326401; doi:10.1186/s12911-026-03492-3)

Example for the Must have, Should have, Could have, Won’t have (MoSCoW) method prioritization framework

**Green: “Must Have”** (Absolutely essential elements that must be included at any cost.)

**Yellow: “Should Have”** (Important elements that should only be omitted with careful consideration.)

**Orange: “Could Have”** (Desirable elements that would ideally be included if resources allow.)

**Red: “Won’t Have”** (Elements that are out-of-scope, unfeasible or actively counterproductive.)

Prioritization of predefined functional requirements


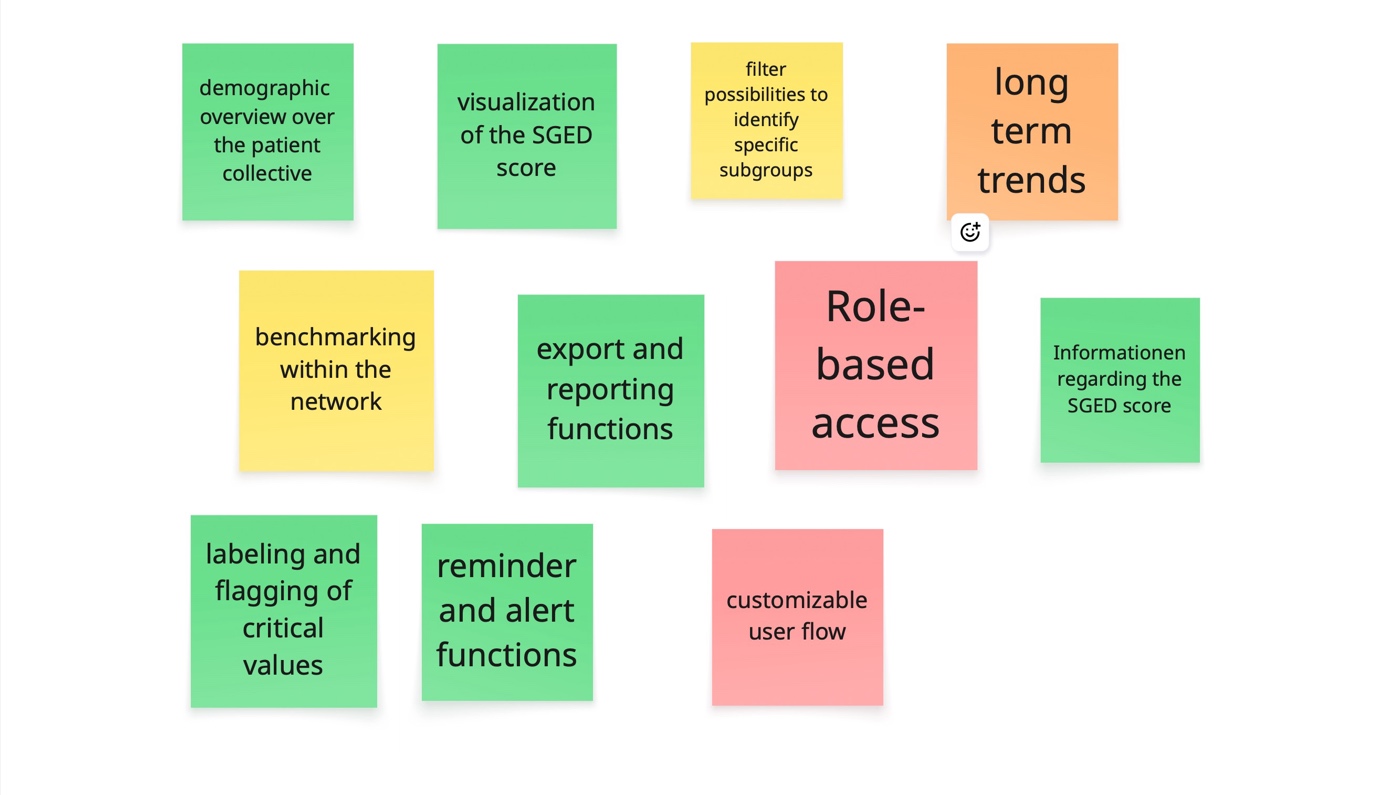


Prioritization of user-generated functional requirements:


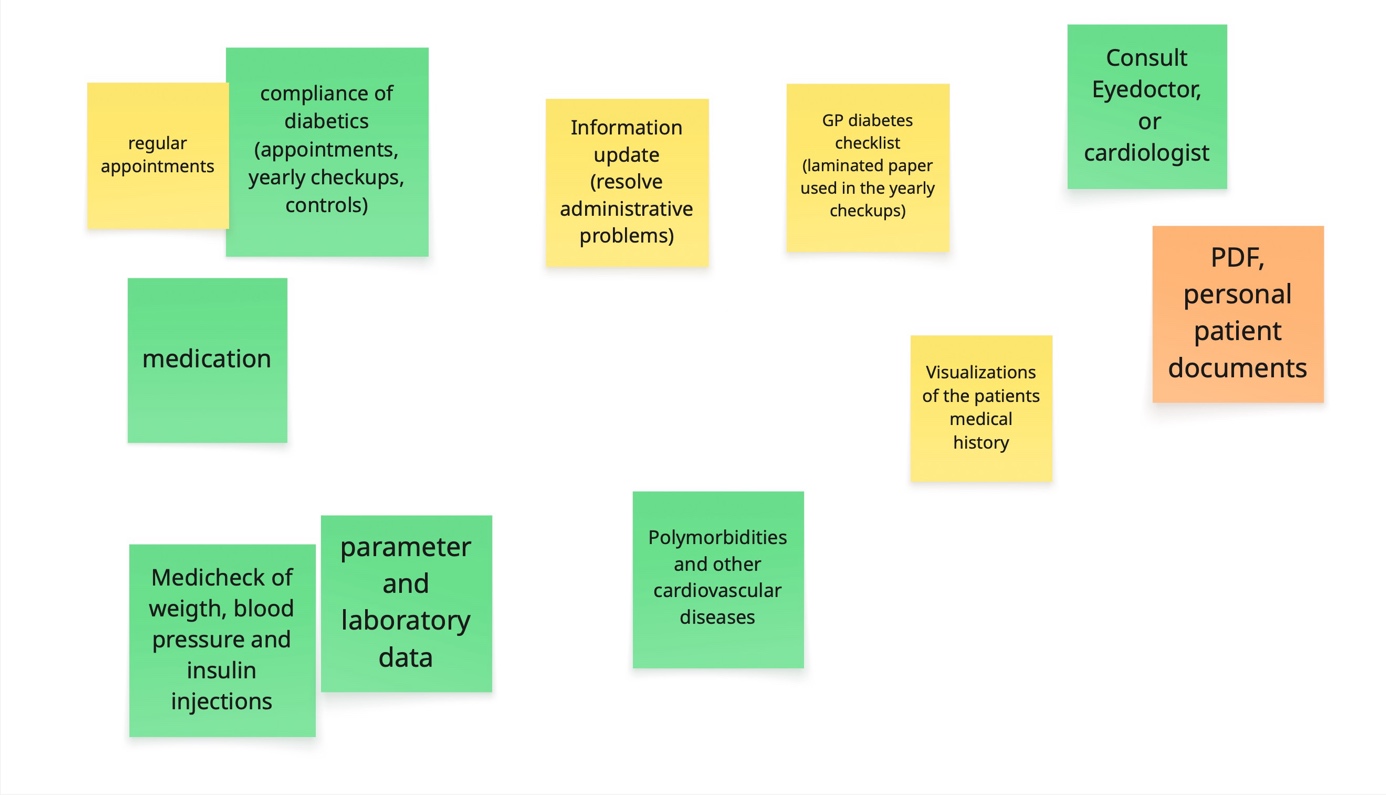

Supplement: Supplementary file 8 — Supplementary Material 8 [file 12911_2026_3492_MOESM8_ESM.docx]
