# Supplementary material for: A care quality dashboard for general practitioners managing patients with diabetes mellitus type 2: user-centered design and prototype evaluation
Source: BMC Med Inform Decis Mak. 2026 May 9;26:234. doi: 10.1186/s12911-026-03492-3 (PMC13326401; doi:10.1186/s12911-026-03492-3)
Supplement: Supplementary file 9 — Supplementary Material 9 [file 12911_2026_3492_MOESM9_ESM.docx]

Screenshots of the SGED Score Dashboard Prototype in German


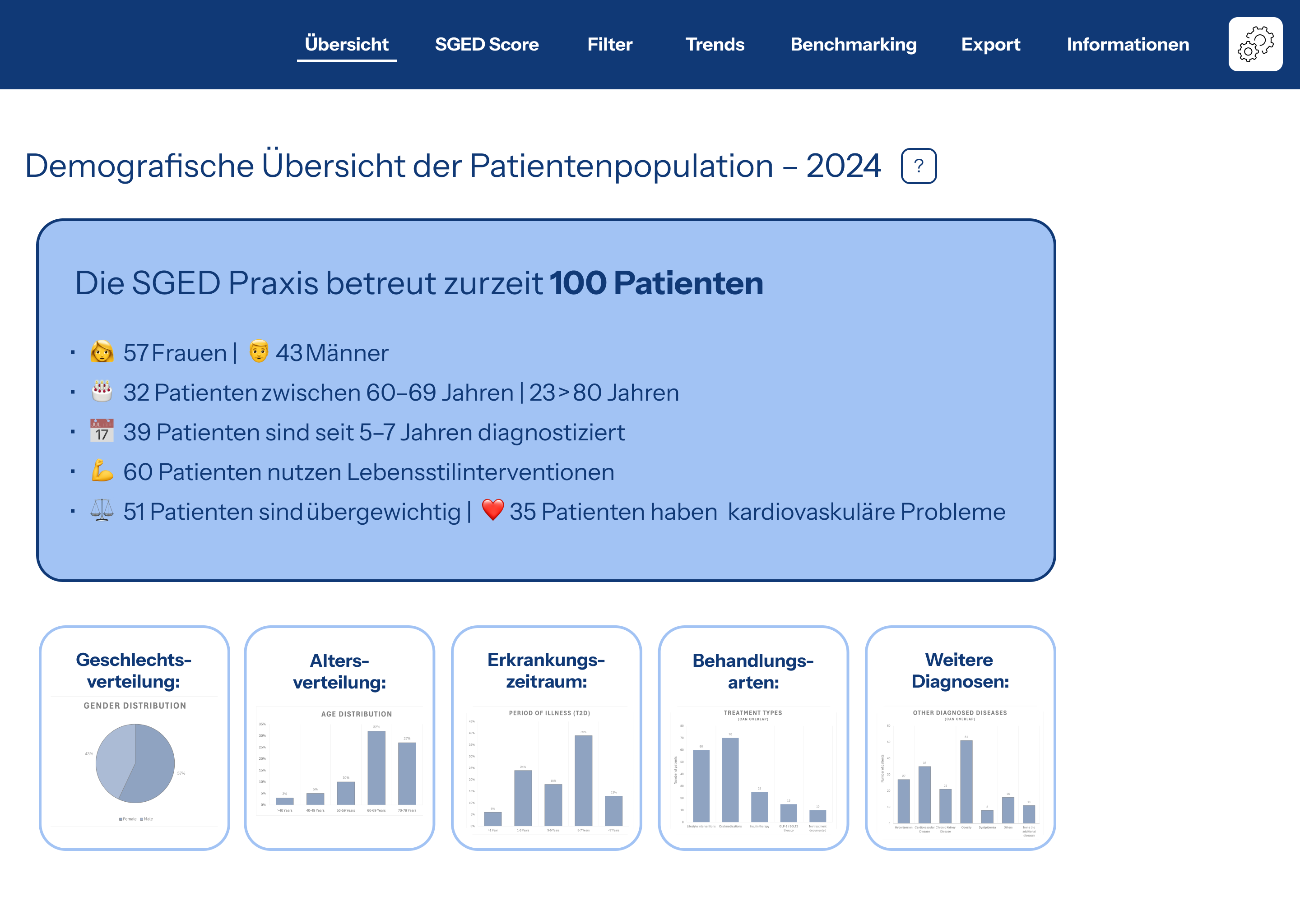


Figure 3: Demographic overview over the patient collective


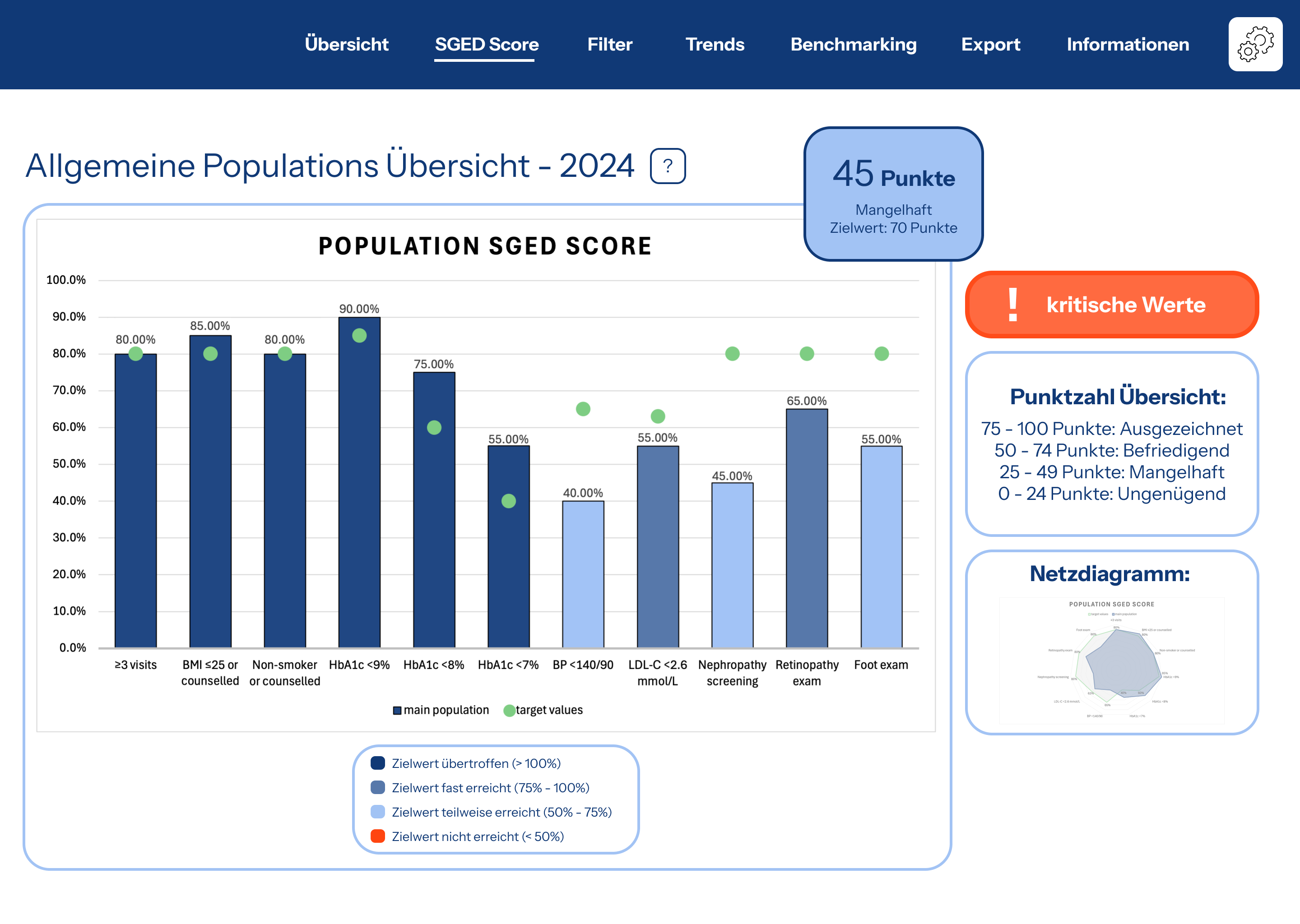


Figure 4: Visualization of the SGED score (main population)


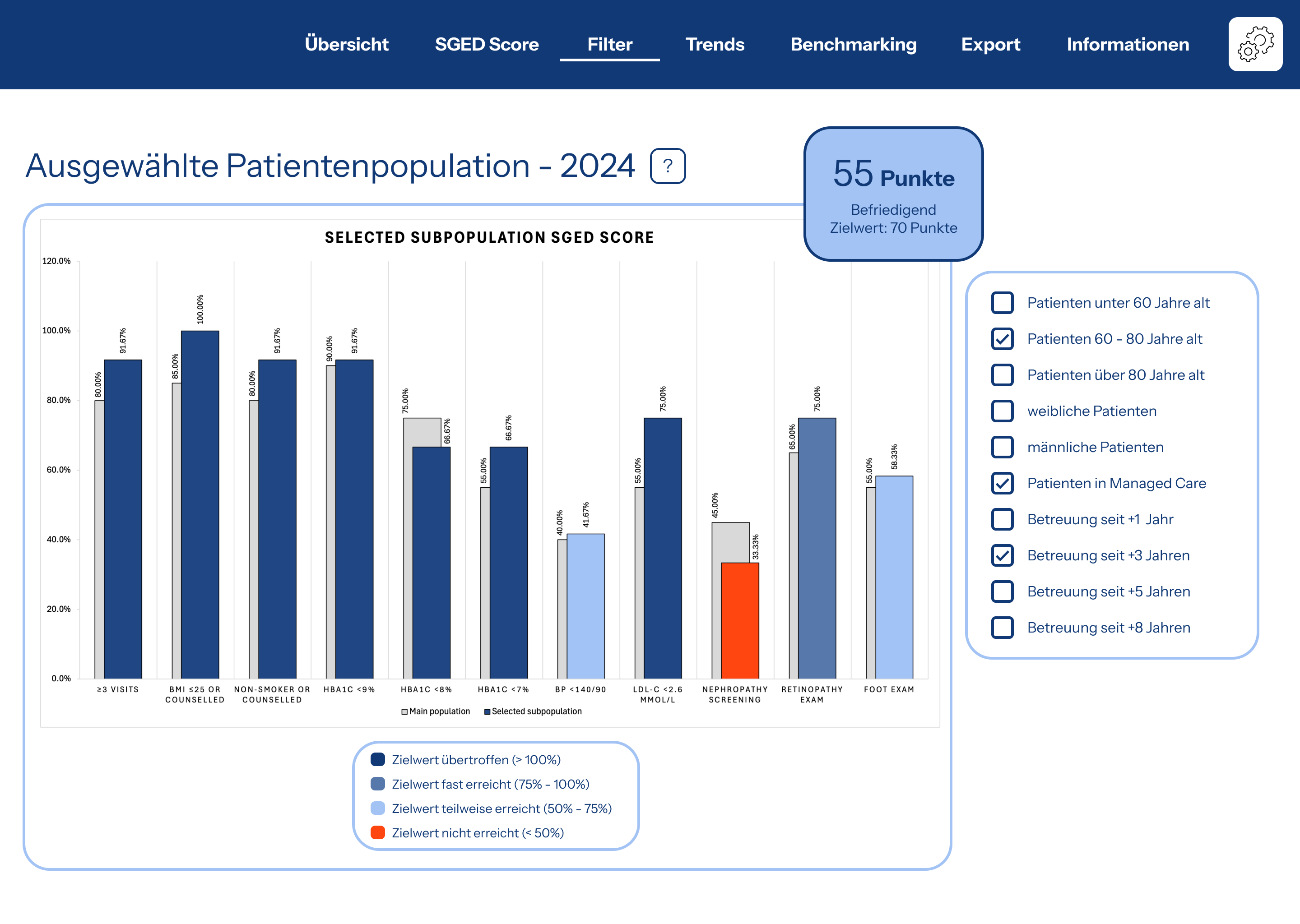


Figure 5: Filter possibilities to identify specific subgroups


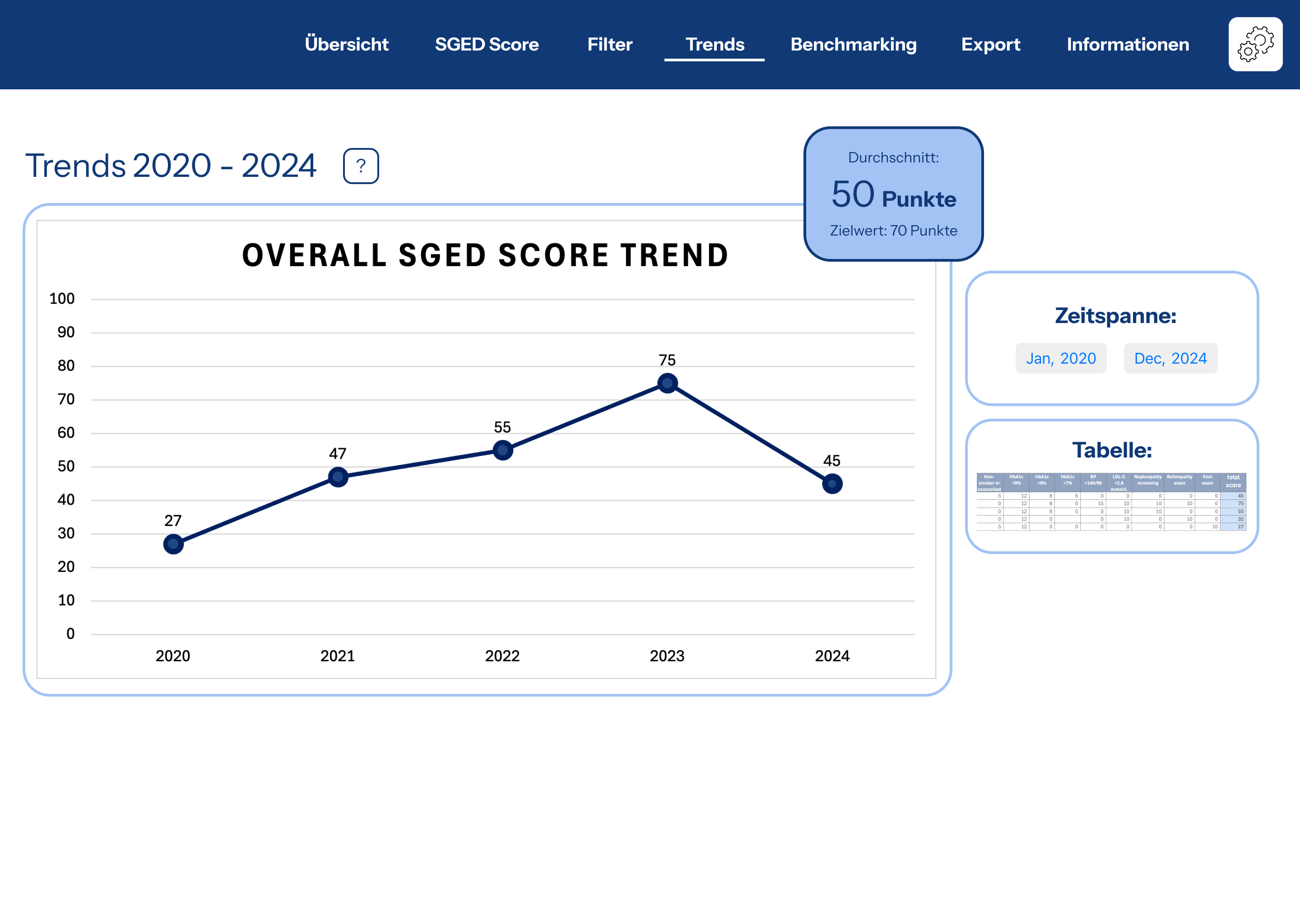


Figure 6: Long term trends


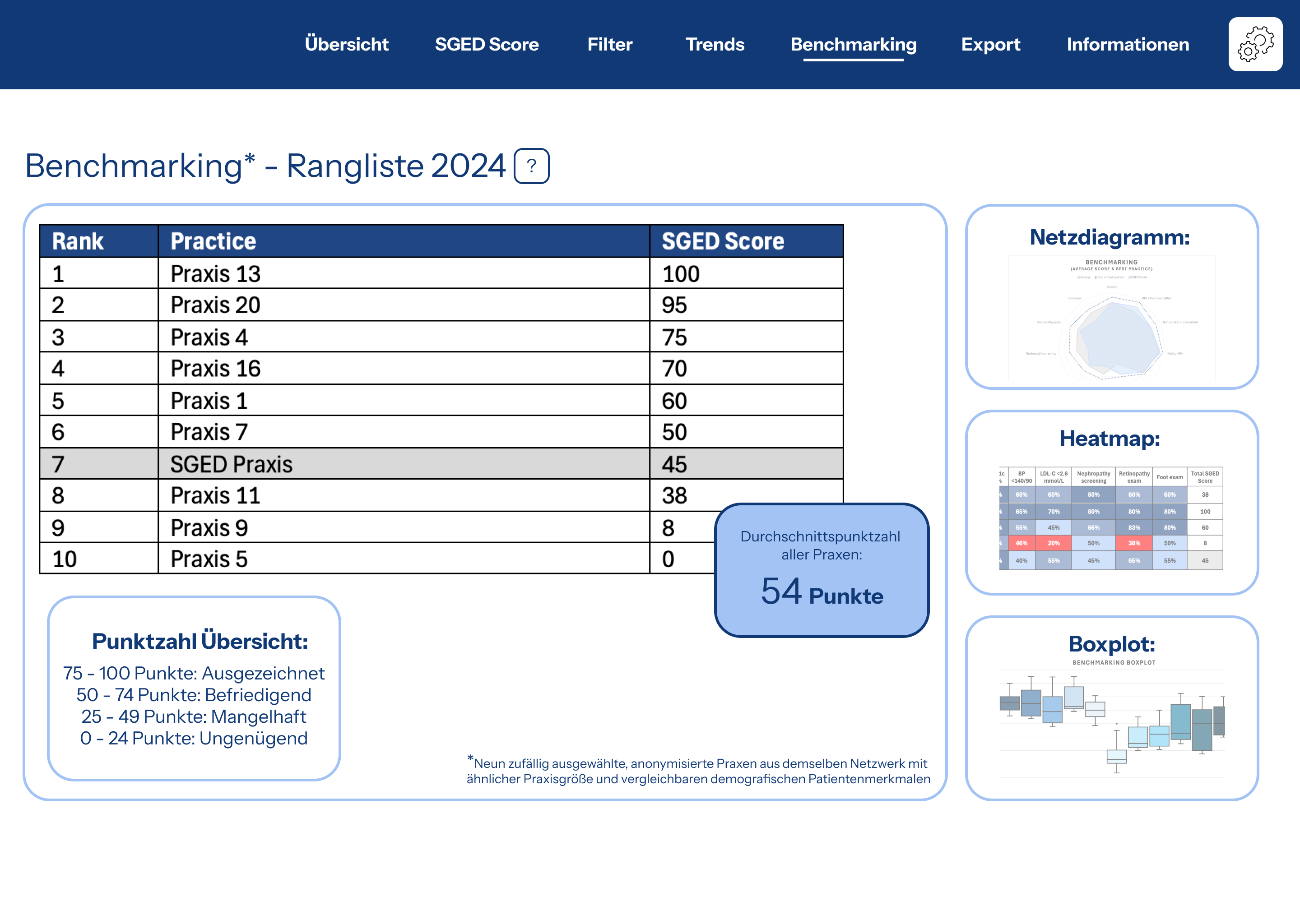


Figure 7: Anonymized benchmarking within the network


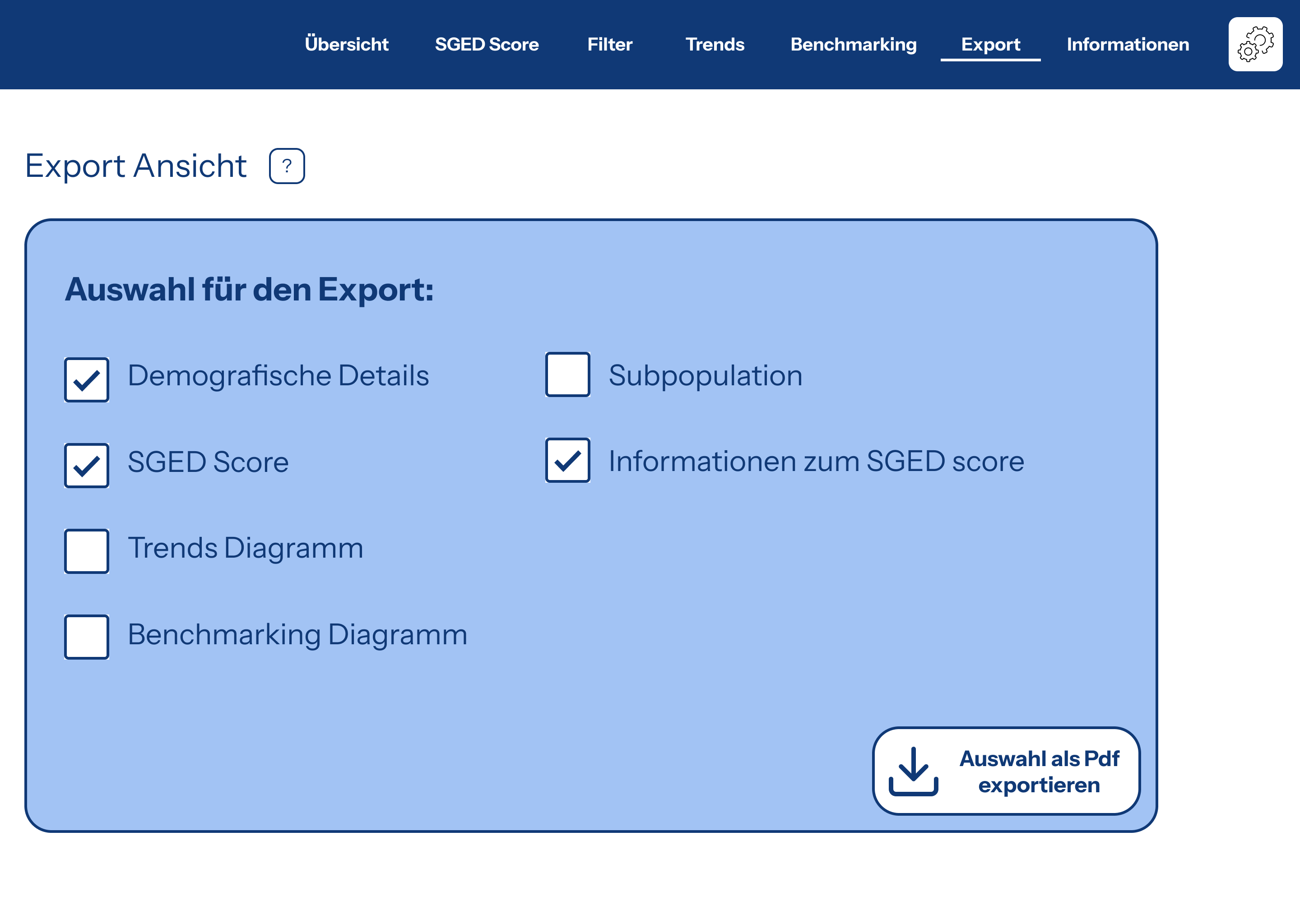


Figure 8: Exporting and reporting function


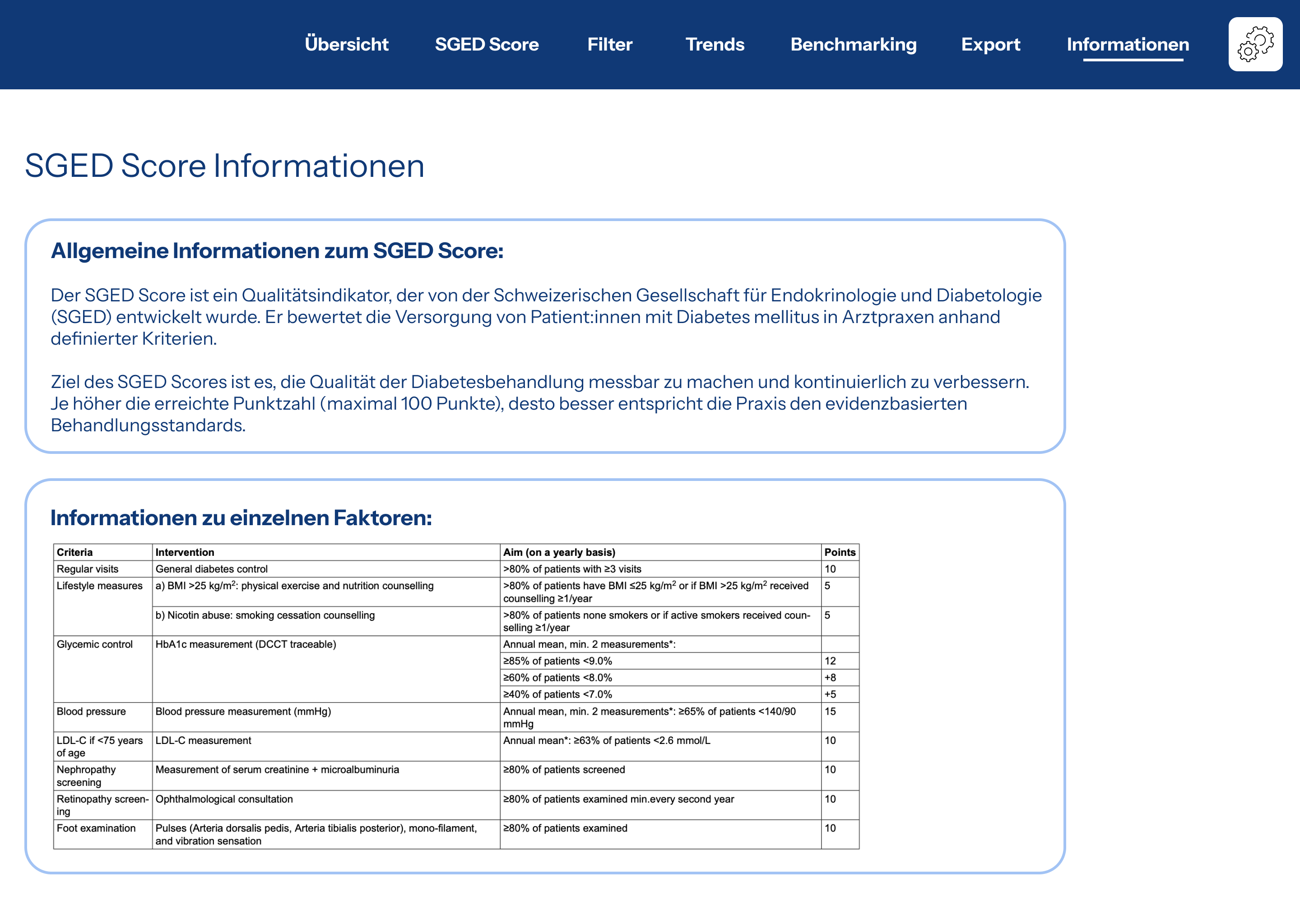


Figure 9: Information regarding the SGED score


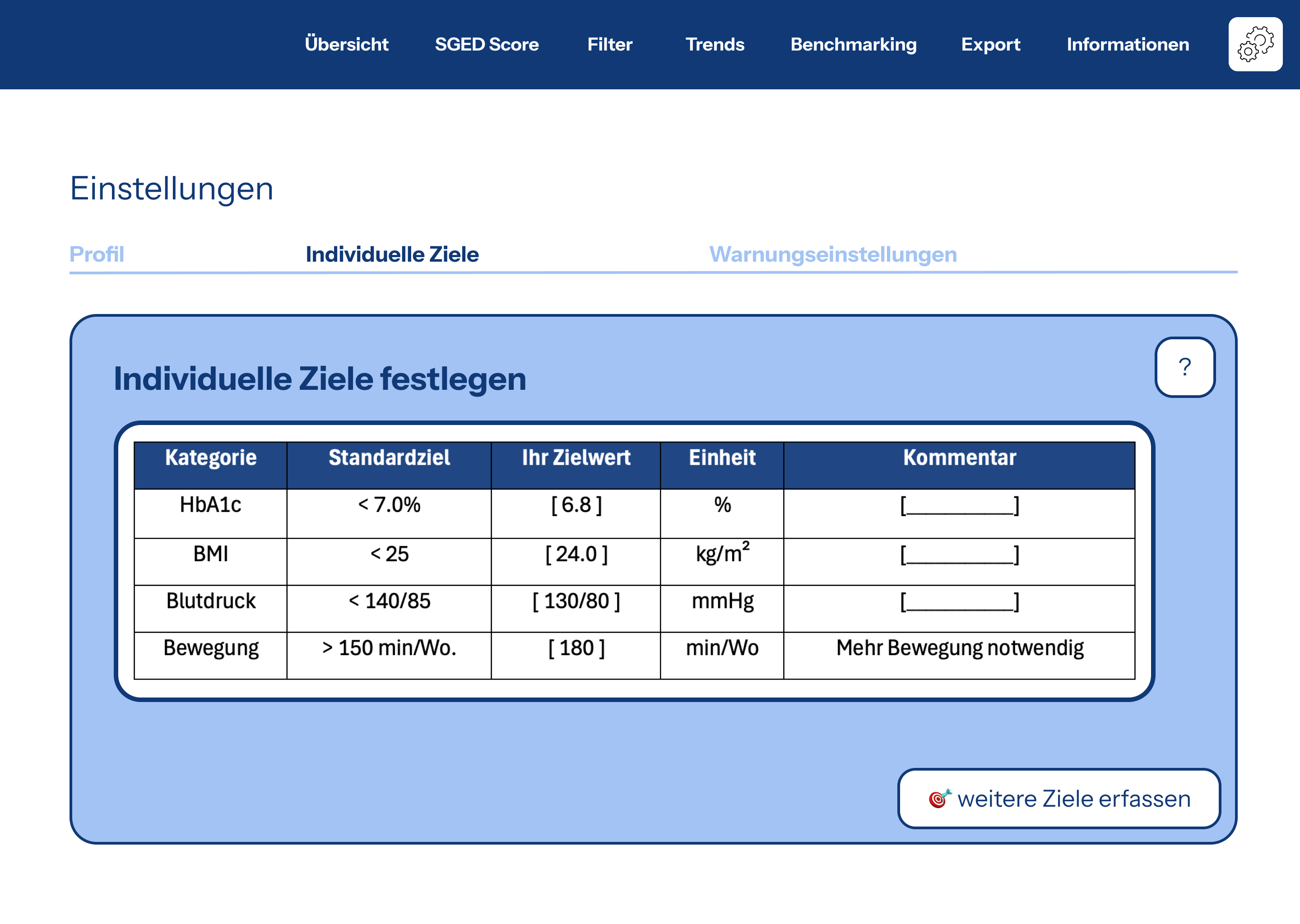


Figure 10: Customizable user flow (setting user specific goals)


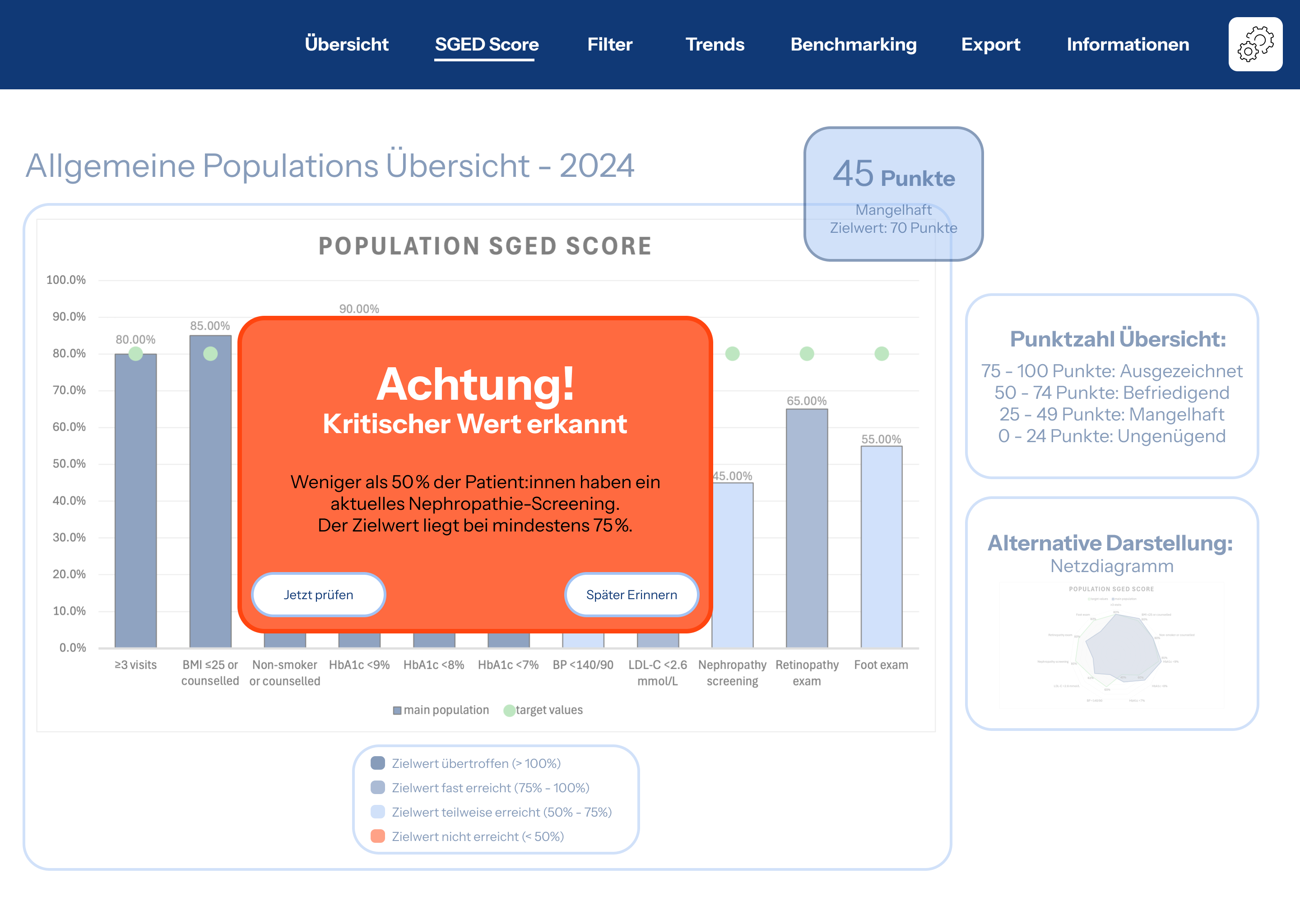


Figure 11: Reminder and alert functions


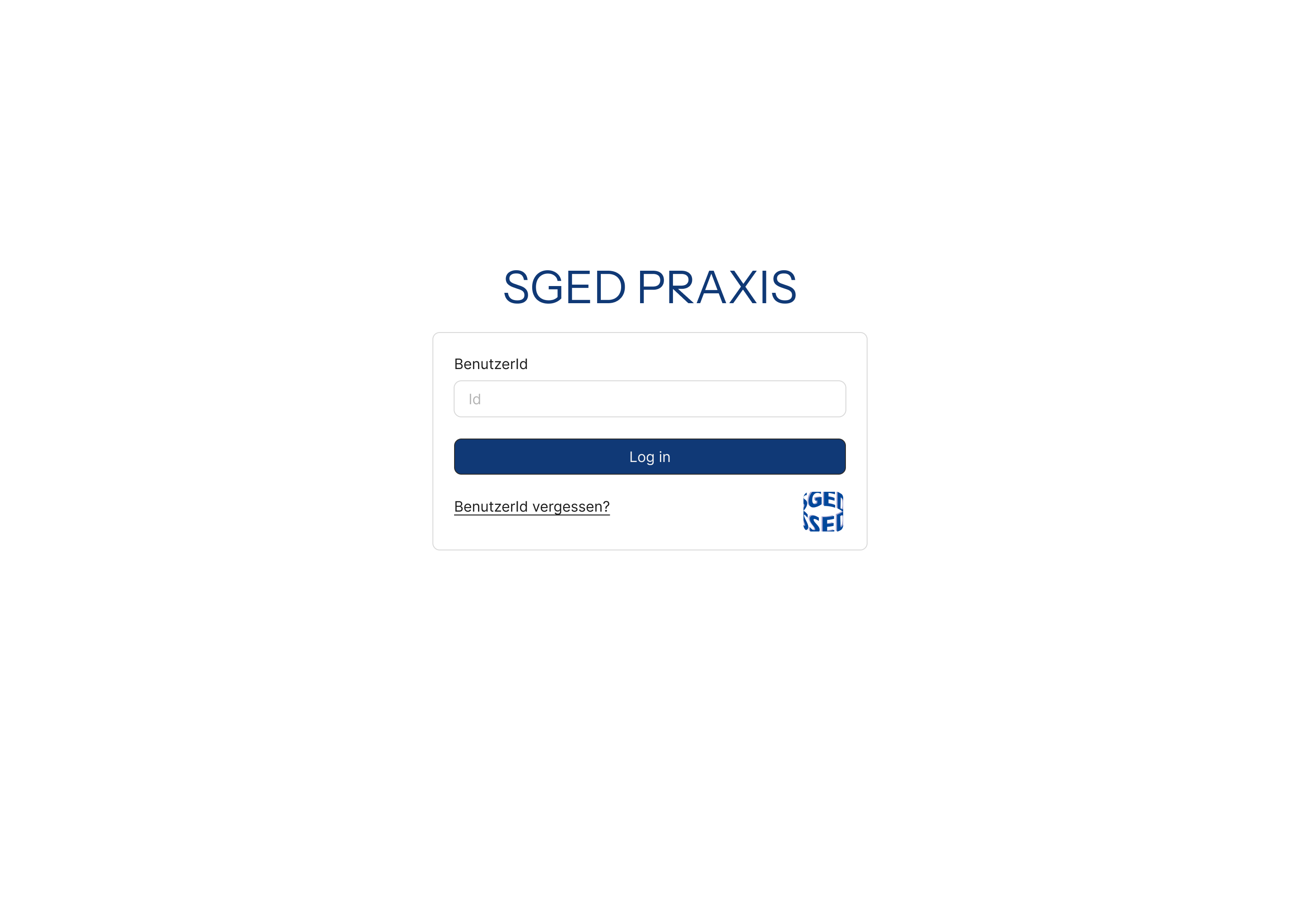


Figure 12: Role-based access
